# Supplementary material for: Osteoblastic Swedish mutant APP expedites brain deficits by inducing endoplasmic reticulum stress-driven senescence
Source: Commun Biol. 2021 Nov 25;4:1326. doi: 10.1038/s42003-021-02843-2 (PMC8617160; doi:10.1038/s42003-021-02843-2)
Supplement: Supplementary file 2 — Supplementary Information [file 42003_2021_2843_MOESM2_ESM.pdf]

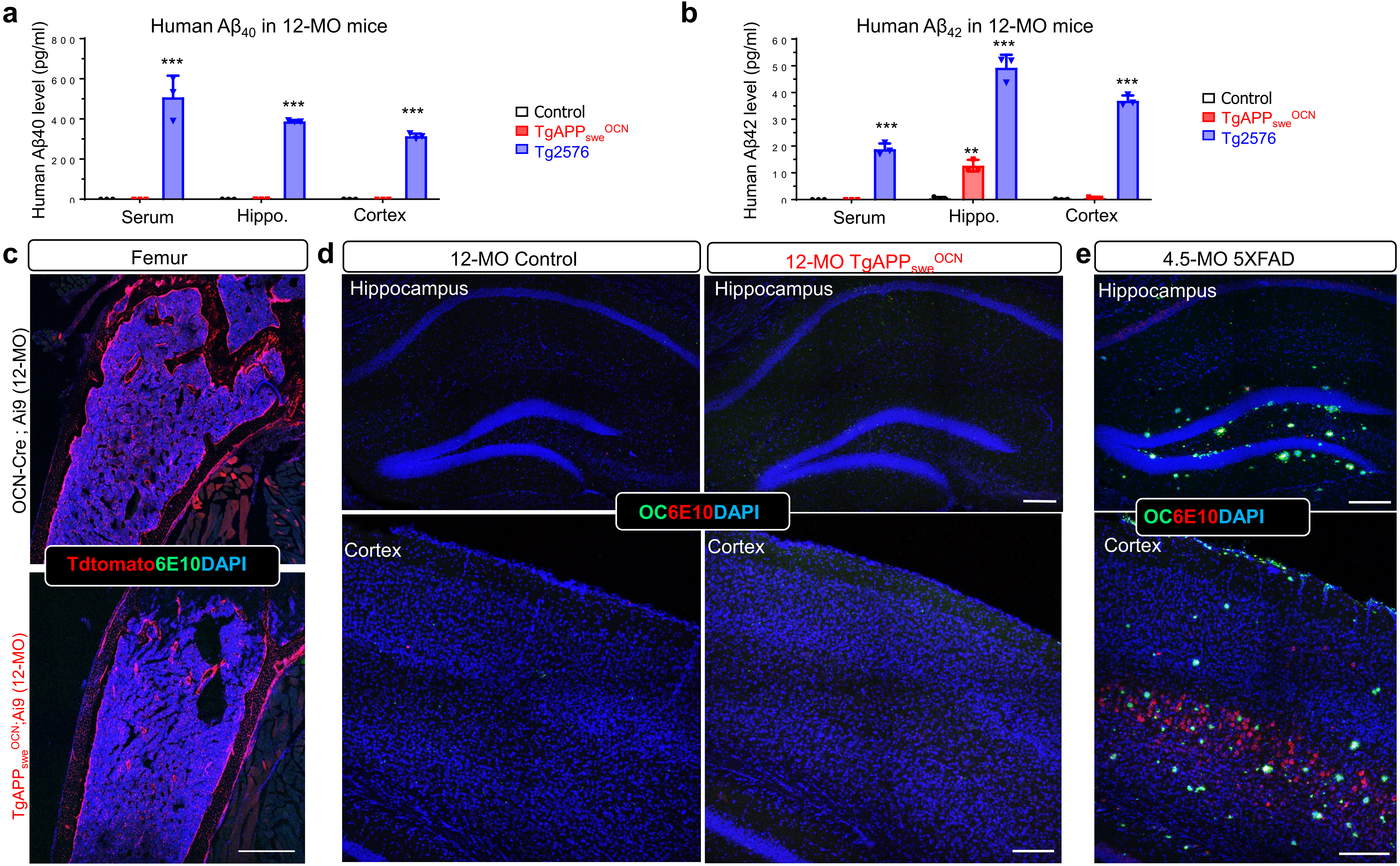

a
Human Aβ40 in 12-MO mice
Human Aβ40 level (pg/ml)
800
600
400
200
0
***
***
***
Serum
Hippo.
Cortex
Control
TgAPPswe OCN
Tg2576
b
Human Aβ42 in 12-MO mice
Human Aβ42 level (pg/ml)
60
50
40
30
20
10
0
***
**
***
***
Serum
Hippo.
Cortex
Control
TgAPPswe OCN
Tg2576
c
Femur
OCN-Cre ; Ai9 (12-MO)
Tdtomato6E10DAPI
TgAPPswe OCN;Ai9 (12-MO)
d
12-MO Control
12-MO TgAPPswe OCN
Hippocampus
Hippocampus
OC6E10DAPI
Cortex
Cortex
e
4.5-MO 5XFAD
Hippocampus
OC6E10DAPI
Cortex

Supplementary Fig. 1: A slight increase of Aβ42, but little to no Aβ40 or Aβ plaque in 12-MO *$TgAPP_{swe}^{OCN}$* hippocampus.

(a-b) Measurement of human Aβ40(**a**) and Aβ42(**b**) levels in serum, hippocampus, and cortex (300*µ*g in total protein) of 12-MO control, *$TgAPP_{swe}^{OCN}$*, and *Tg2576* mice by Elisa assay. **(c)** Representative images of bone sections from 12-MO control *(OCN-Cre; Ai9*) and *$TgAPP_{swe}^{OCN}$; Ai9* mice coimmunostained with 6E10 (green) and DAPI (blue). Scale bar, 500*µ*m. (**d**) Representative images of hippocampal and cortical brain sections from 12-MO control *(LSL-APP_{swe}*) and *$TgAPP_{swe}^{OCN}$* coimmunostained with 6E10 (red), anti-amyloid fibrils OC (green), and DAPI (blue). Scale bars, 200*µ*m. **(e)** Representative images of hippocampal and cortical brain sections from 4.5-MO 5XFAD coimmunostained with 6E10 (red), anti-amyloid fibrils OC (green), and DAPI (blue). Scale bars, 200*µ*m. In (a-b) all values were presented as mean ± SD. n=3 mice in each group. $^{**}p < 0.01$, $^{***}p < 0.001$. One-way ANOVA followed by Tukey post hoc test.

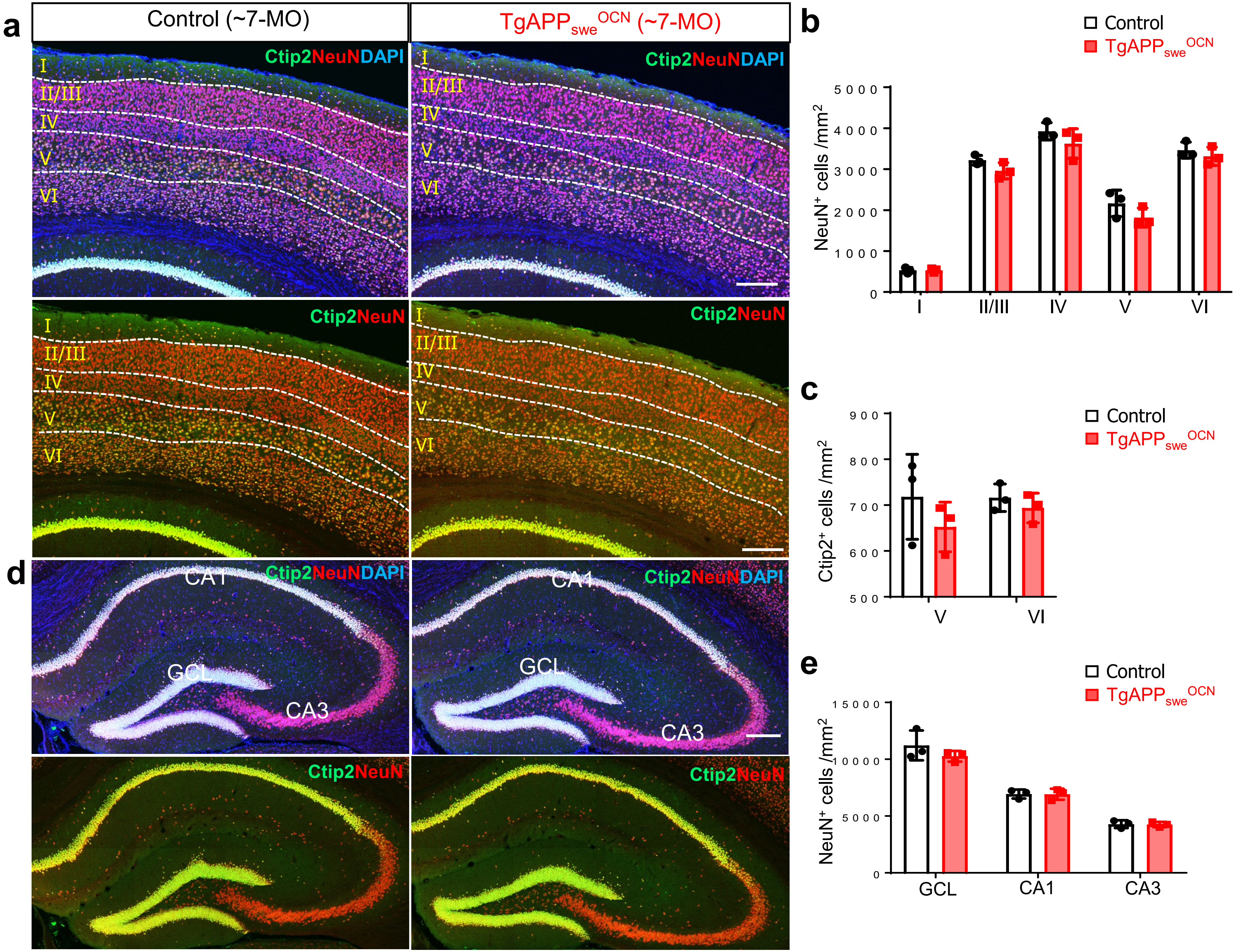

a
Control (~7-MO)
TgAPPswe OCN (~7-MO)
Ctip2NeuNDAPI
Ctip2NeuN
I
II/III
IV
V
VI
b
Control
TgAPPswe OCN
NeuN+ cells /mm2
0
1000
2000
3000
4000
5000
I
II/III
IV
V
VI
c
Ctip2+ cells /mm2
500
600
700
800
900
V
VI
d
CA1
GCL
CA3
e
NeuN+ cells /mm2
0
5000
10000
15000
GCL
CA1
CA3

Supplementary Fig. 2: Normal neuron distribution pattern and densities in the cortex and hippocampus of *$TgAPP_{swe}^{OCN}$* mice.

(**a**)Representative images of cortical brain sections from ~7-MO control and *$TgAPP_{swe}^{OCN}$* coimmunostained with Ctip2 (green), NeuN (red), and DAPI (blue). Scale bar, 200*μ*m. **(b-c)** Quantification analyses of NeuN cell densities in different cortical layers (**b**) and Ctip2 cell densities in V and VI layer (**c**) in **a**. **(d)** Representative images of dorsal hippocampal sections from ~7-MO Control and *$TgAPP_{swe}^{OCN}$* coimmunostained with Ctip2 (green), NeuN (red), and DAPI (blue). Scale bar, 200*μ*m. **(e)** Quantification analyses of NeuN cell densities in GCL, CA1, CA3 in **d**. All data were presented as mean ± SD (n=3), and no significant difference was detected by Mann-Whitney U test.

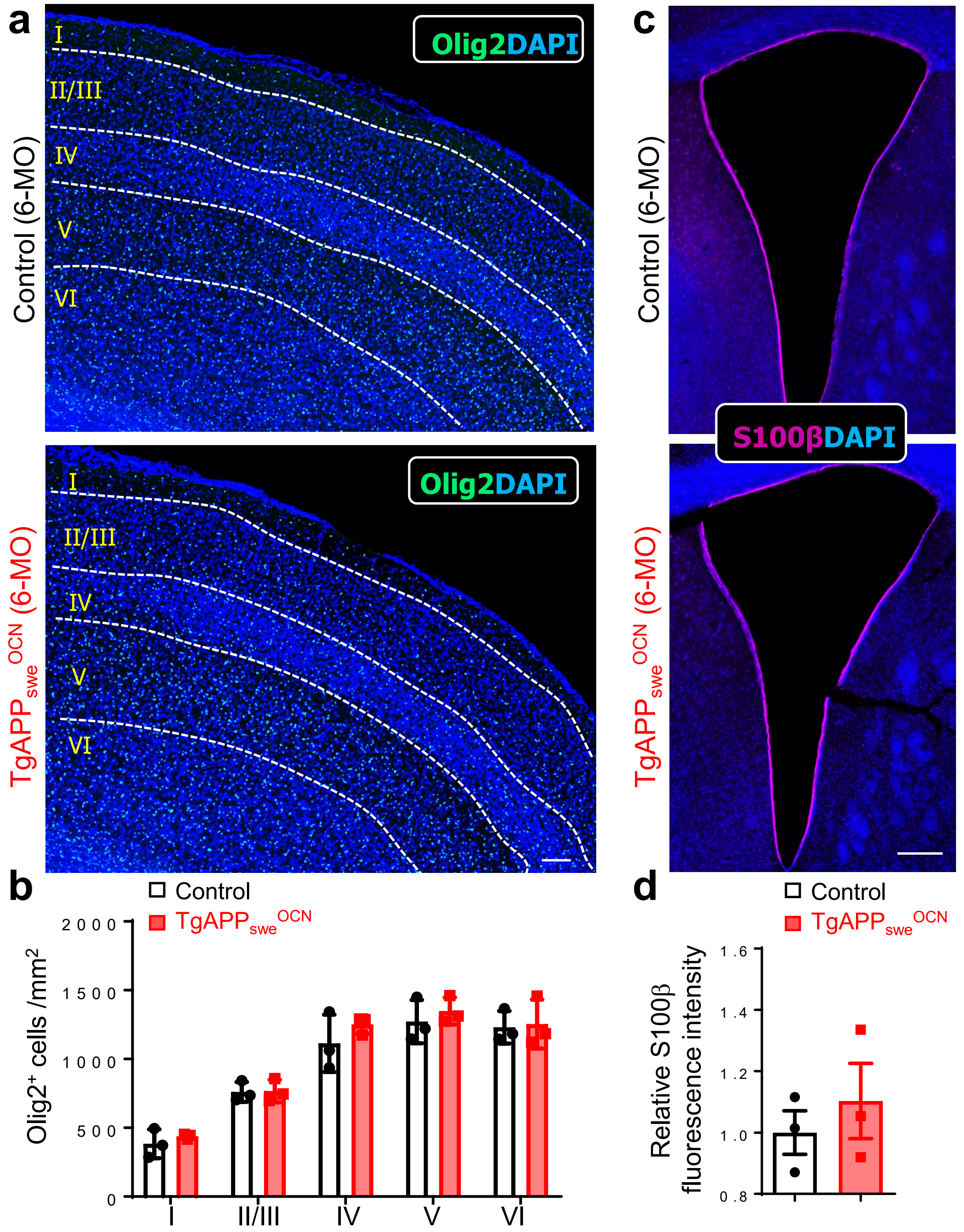
a
Olig2DAPI
I
II/III
IV
V
VI
Control (6-MO)
TgAPP$_{swe}^{OCN}$ (6-MO)
b
Control
TgAPP$_{swe}^{OCN}$
Olig2$^+$ cells /mm^2
2000
1500
1000
500
0
I
II/III
IV
V
VI
c
S100βDAPI
Control (6-MO)
TgAPP$_{swe}^{OCN}$ (6-MO)
d
Control
TgAPP$_{swe}^{OCN}$
Relative S100β fluorescence intensity
1.6
1.4
1.2
1.0
0.8

Supplementary Fig. 3: Normal Olig2⁺ oligodrocytes and S100β⁺ ependymal cells in 6-MO $TgAPP_{swe}^{OCN}$ mice.

(a) Representative images of cortical brain sections from 6-MO control and $TgAPP_{swe}^{OCN}$ mice coimmunostained with Olig2 (green) and DAPI (blue). Scale bar, 100*μ*m. **(b)** Quantification analyses of cortical $Olig2^+$ cell densities in **a**. **(c)** Representative images of subventricular zone sections from 6-MO control and $TgAPP_{swe}^{OCN}$ mice coimmunostained with S100β (magenta), and DAPI (blue). Scale bar, 100*μ*m. **(d)** Quantification analyses of $S100\beta^+$ fluorescence intensity in **c**. All data were presented as mean ± SD (n = 3), and no significant difference was detected, by Mann-Whitney U test.

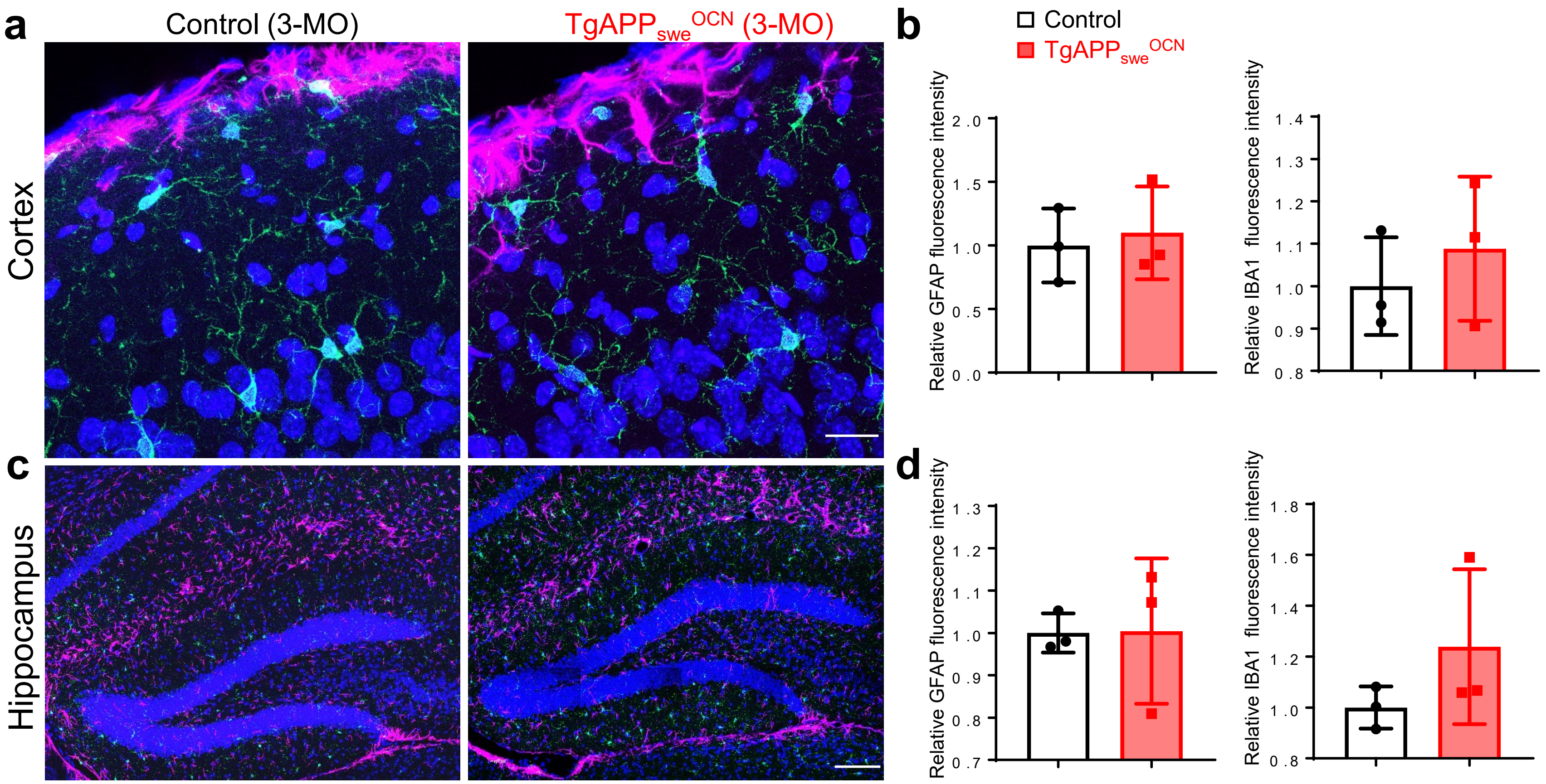
a
Control (3-MO)
TgAPPswe OCN (3-MO)
Cortex
c
Hippocampus
b
Control
TgAPPswe OCN
Relative GFAP fluorescence intensity
Relative IBA1 fluorescence intensity
d
Relative GFAP fluorescence intensity
Relative IBA1 fluorescence intensity

Supplementary Fig. 4: Normal GFAP⁺ astrocytes and IBA1⁺ microglial cells in 3-MO *TgAPP*$_{swe}^{OCN}$ mice.

(**a**) Representative images of cortical brain sections from 3-MO control and *TgAPP*$_{swe}^{OCN}$ mice coimmunostained with IBA1 (green), GFAP (magenta), and DAPI (blue). Scale bar, 20μm. **(b)** Quantification analyses of cortical GFAP and IBA1 fluorescence intensity data in **a**. **(c)** Representative images of hippocampal sections from 3-MO control and *TgAPP*$_{swe}^{OCN}$ mice coimmunostained with IBA1 (green), GFAP (magenta), and DAPI (blue). Scale bar, 100μm. **(d)** Quantification analyses of hippocampal GFAP and IBA1 fluorescence intensity data in **c**. All the data were presented as mean ± SD (n = 3 mice per genotype group), and no significant difference was detected by Mann-Whitney U test.

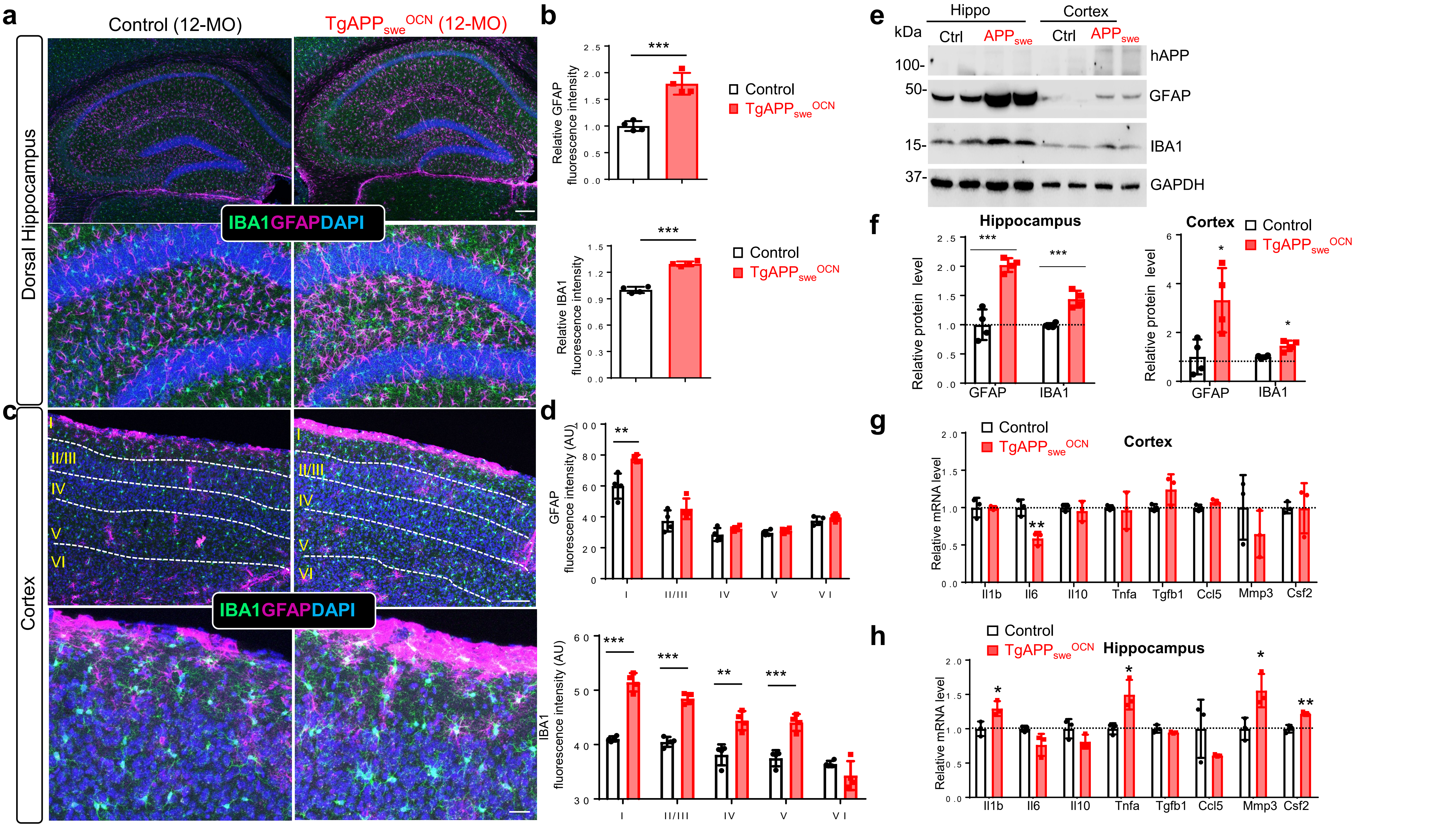

a
Control (12-MO)
TgAPPswe OCN (12-MO)
Dorsal Hippocampus
IBA1GFAPDAPI
b
***
Relative GFAP fluorescence intensity
Control
TgAPPswe OCN
Relative IBA1 fluorescence intensity
c
Cortex
I
II/III
IV
V
VI
d
GFAP fluorescence intensity (AU)
IBA1 fluorescence intensity (AU)
e
kDa
Hippo
Cortex
Ctrl
APPswe
hAPP
GFAP
IBA1
GAPDH
100-
50-
15-
37-
f
Hippocampus
Cortex
Relative protein level
g
Relative mRNA level
Il1b
Il6
Il10
Tnfa
Tgfb1
Ccl5
Mmp3
Csf2
h

Supplementary Fig.5: Elevated reactive astrocytes, microglial cells, and inflammatory cytokines in 12-MO $TgAPP_{swe}^{OCN}$ hippocampus.

(a, c) Representative images of hippocampal (**a**) and cortical (**c**) brain sections from 12-MO control ($LSL\text{-}APP_{swe}$) and $TgAPP_{swe}^{OCN}$ coimmunostained with IBA1 (green), GFAP (magenta), and DAPI (blue). Scale bars: 200μm (upper) and 20μm (lower). **(b, d)** Quantification analyses of GFAP and IBA1 fluorescence intensity data in **a** and **c**, respectively. (**e**) Western blot analysis of GFAP and IBA1 protein expression in cortex and hippocampus from 12-MO Control and $TgAPP_{swe}^{OCN}$ mice. GAPDH was used as a loading control. **(f)** Quantification analysis of the data in **e**. **(g-h)** Real-time PCR (RT-PCR) analysis of indicated gene expression in 12-MO control ($LSL\text{-}APP_{swe}$) and $TgAPP_{swe}^{OCN}$ cortex and hippocampus. All quantification data were presented as mean ± SD (n = 4 for **b**, **d**, **f**, and n= 3 for **g-h**). *$p<0.05$, **$p<0.01$, and ***$p < 0.001$. Student's t test was used in **b**, **d** and **f**, and Mann-Whitney U test was used in **g** and **h**.

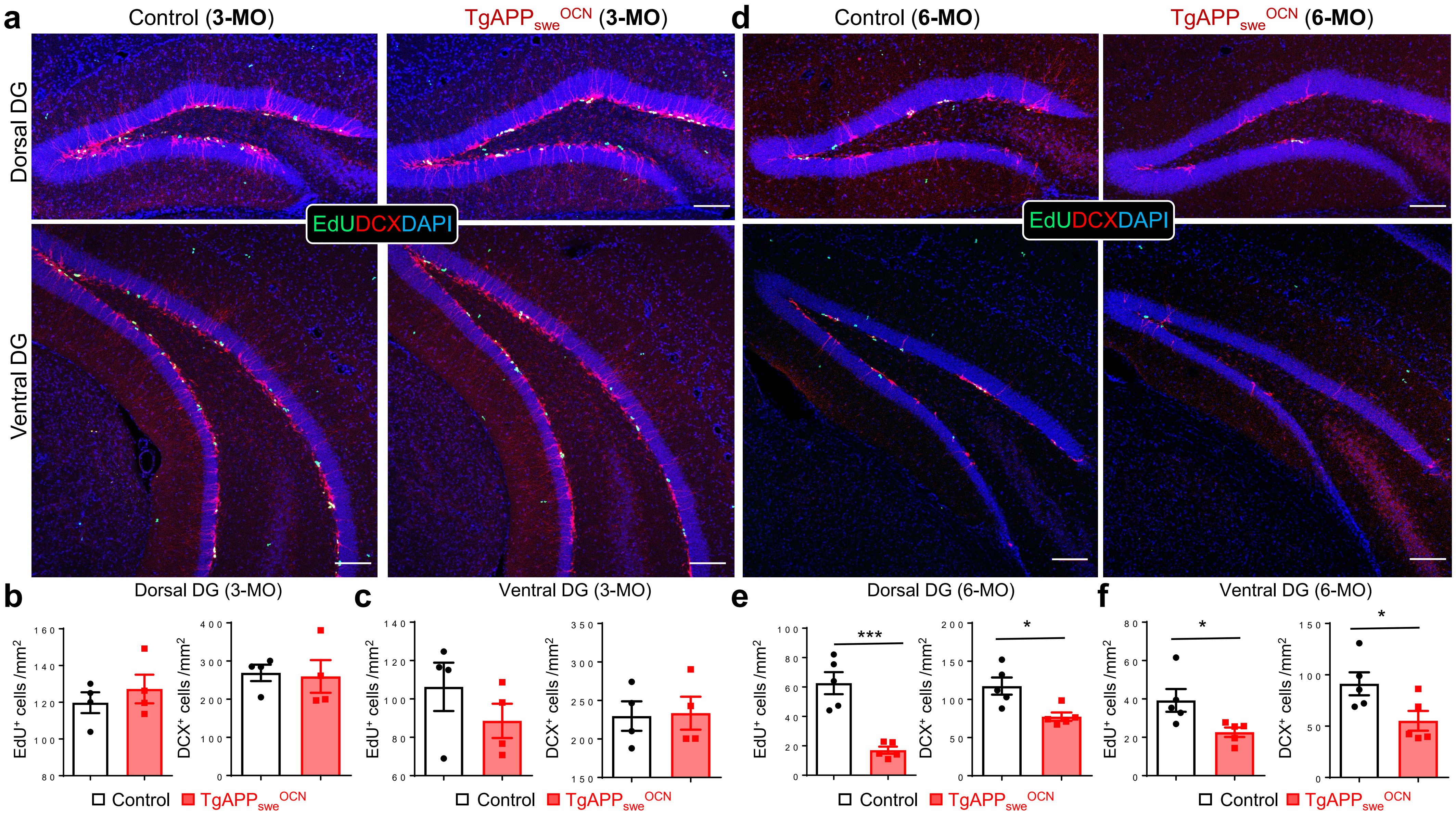
a
Control (3-MO)
TgAPPswe OCN (3-MO)
d
Control (6-MO)
TgAPPswe OCN (6-MO)
Dorsal DG
Ventral DG
EdUDCXDAPI
EdUDCXDAPI
b
Dorsal DG (3-MO)
c
Ventral DG (3-MO)
e
Dorsal DG (6-MO)
f
Ventral DG (6-MO)
EdU+ cells /mm2
DCX+ cells /mm2
***
*
Control
TgAPPswe OCN

Supplementary Fig. 6: Age-dependent impairment of hippocampus DG neurogenesis in $TgAPP_{swe}^{OCN}$ mice.

3- and 6-MO control (*LSL-APPswe*) and $TgAPP_{swe}^{OCN}$ mice were given four intraperitoneal injections of EdU (50 mg/kg/time, 1 time/4 h) within 12 h, and 12 hours after the last injection, mice were euthanized, and brain sections were subjected for co-immunostaining analyses using indicated antibodies. (**a**) Representative images of dorsal and ventral DG from 3-MO control and $TgAPP_{swe}^{OCN}$ mice. Scale bar, 100*μ*m. **(b-c)** Quantification analyses of EdU^+ and DCX^+ cell densities in dorsal (**b**) and ventral DG (**c**) of data in **a**. (**d**) Representative images of dorsal and ventral DG from 6-MO control and $TgAPP_{swe}^{OCN}$ mice. Scale bar, 100*μ*m. **(e-f)** Quantification analyses of EdU^+ and DCX^+ cell densities in dorsal (**e**) and ventral DG (**f**) of data in **d**. All data were presented as mean ± SD (n =4-5 mice per genotype group). *p<0.05, ***p < 0.001. Student's t test.

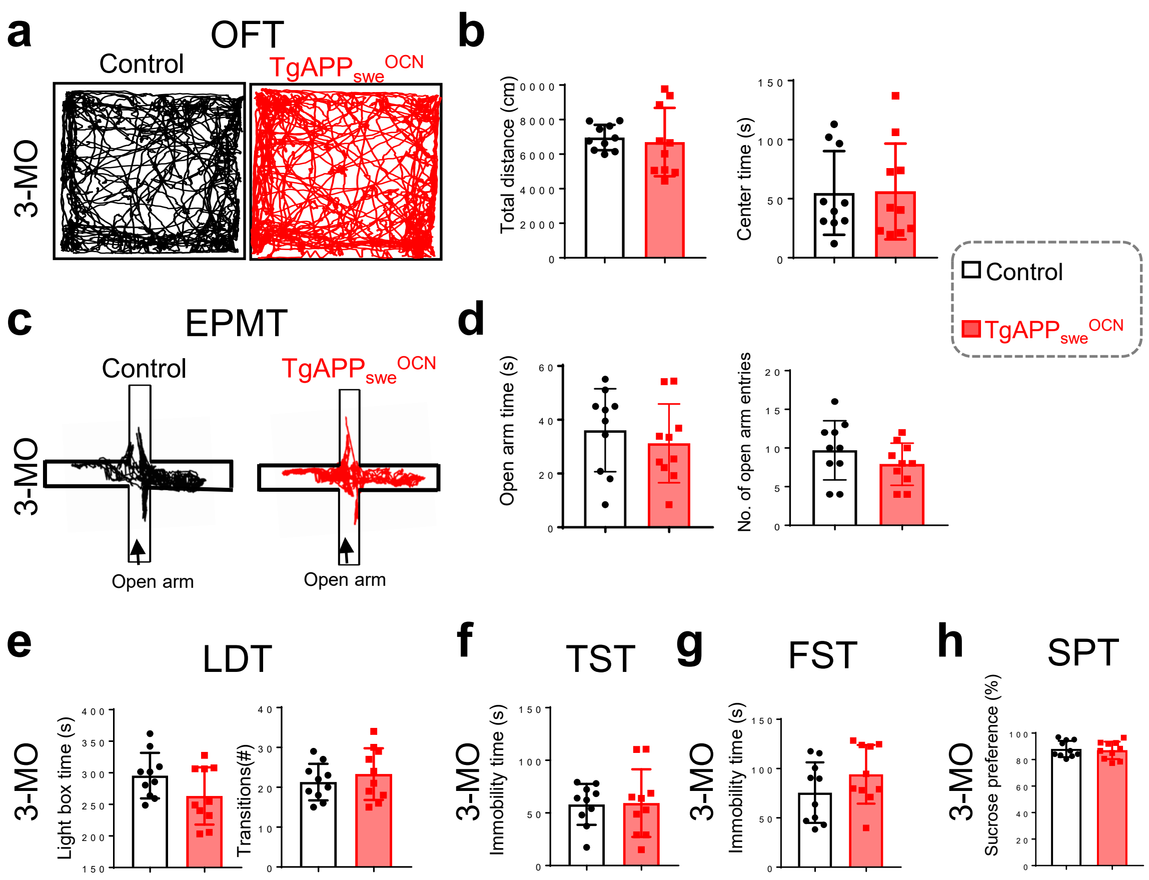
a
OFT
Control
TgAPPswe OCN
3-MO
b
Total distance (cm)
Center time (s)
Control
TgAPPswe OCN
c
EPMT
Control
TgAPPswe OCN
3-MO
Open arm
Open arm
d
Open arm time (s)
No. of open arm entries
e
LDT
3-MO
Light box time (s)
Transitions(#)
f
TST
3-MO
Immobility time (s)
g
FST
3-MO
Immobility time (s)
h
SPT
3-MO
Sucrose preference (%)

Supplementary Fig. 7: Undetectable anxiety- and depression-like behaviors in 3-MO *TgAPPswe^{OCN}* mice.

(a-b) OFT: Representative tracing images(**a**) and quantifications of total distance and center duration time (**b**). **(c-d)** EPMT: Representative tracing images (**c**) and quantifications of open arm duration time and entries **(d)**. **(e) LDT:** Quantifications of the time spent in the light room and the number of transitions into the light room. **(f)** TST, **(g)** FST, and **(h)** SPT quantification data. In all tests, 3-MO control (*LSL-APP$_{swe}$*) and *TgAPP$_{swe}^{OCN}$* mice (males) were examined. All data were shown as mean ± SD (n= 10 mice per genotype). No significant difference was detected by student's t test.

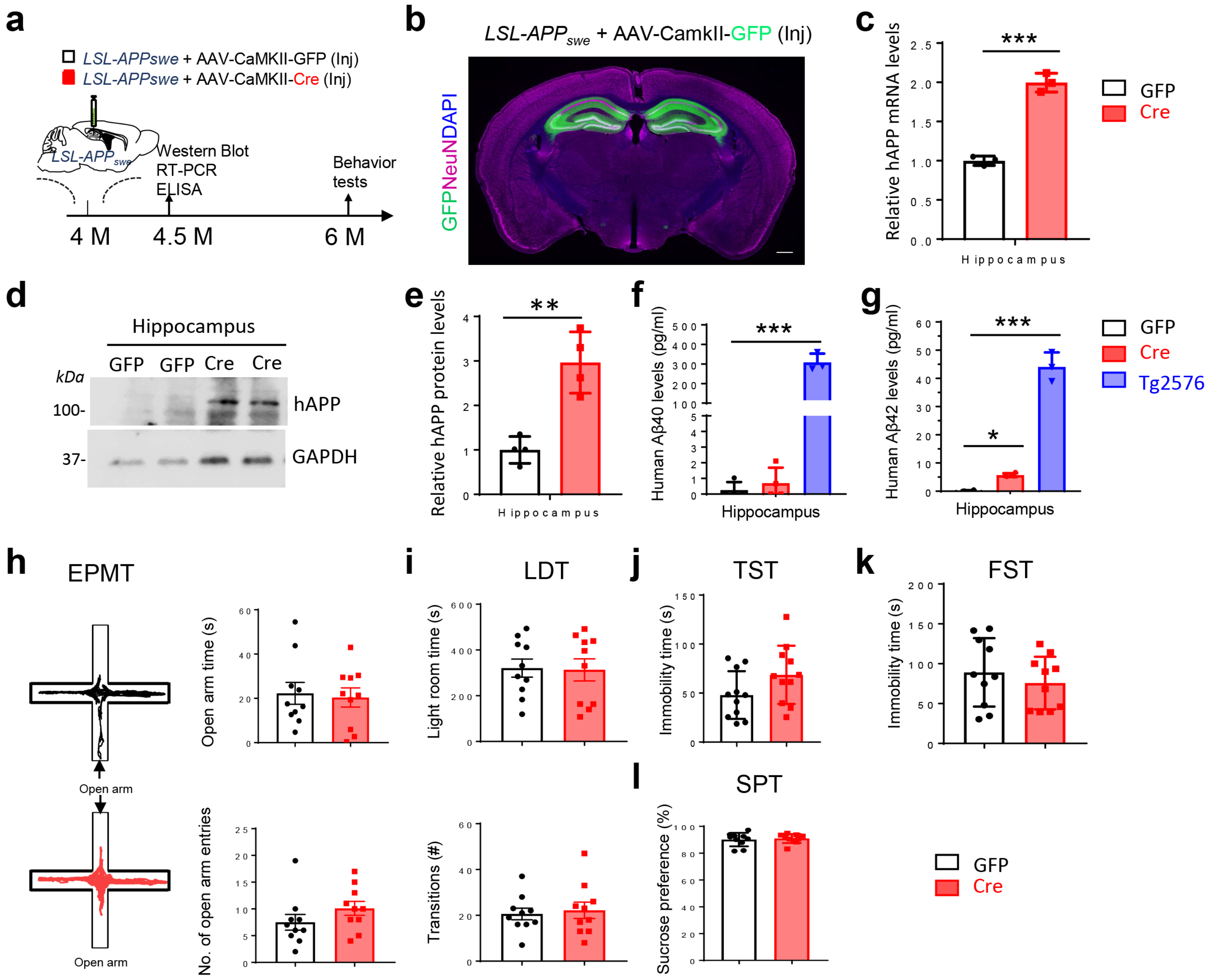
a
LSL-APPswe + AAV-CaMKII-GFP (Inj)
LSL-APPswe + AAV-CaMKII-Cre (Inj)
LSL-APPswe
Western Blot
RT-PCR
ELISA
Behavior tests
4 M
4.5 M
6 M
b
LSL-APPswe + AAV-CamkII-GFP (Inj)
GFPNeuNDAPI
c
Relative hAPP mRNA levels
Hippocampus
GFP
Cre
d
Hippocampus
GFP GFP Cre Cre
kDa
100-
37-
hAPP
GAPDH
e
Relative hAPP protein levels
Hippocampus
f
Human Aβ40 levels (pg/ml)
Hippocampus
g
Human Aβ42 levels (pg/ml)
Hippocampus
GFP
Cre
Tg2576
h
EPMT
Open arm
Open arm
Open arm time (s)
No. of open arm entries
i
LDT
Light room time (s)
Transitions (#)
j
TST
Immobility time (s)
k
FST
Immobility time (s)
l
SPT
Sucrose preference (%)
GFP
Cre

Supplementary Fig. 8: A slight increase of Aβ_{42}, but undetectable anxiety- and depression-like behaviors in *LSL-APP$_{swe}$* mice injected with AAV-CamkII-Cre into their dorsal DGs.

(a) Schematic diagram of experimental design for western blot, RT-PCR, Elisa assay (2 week after injection) and behavioral tests (2 month after injection) in *LSL-APPswe* mice injected with AAV9-CamkII-GFP (as a control) or AAV9-CamkII-Cre. **(b)** Representative image of GFP expression in DG neurons in *LSL-APP$_{swe}$* mice injected with AAV9-CamkII-GFP. Scale bar, 200μm. (**c**) Real-time PCR (RT-PCR) analysis of relative *hAPP* gene expression in hippocampus of indicated groups. ***$p < 0.001$. n=3, Mann-Whitney U test. (**d**) Western blot analysis of hAPP expression in hippocampus from *LSL-APP$_{swe}$* mice injected with AAV-CamkII-GFP or AAV-CamkII-Cre, GAPDH was used as a loading control. **(e)** Quantification analysis of the data in **d**, **$p < 0.01$. n=4, student's t test. **(f-g)** Measurement of human Aβ40 and Aβ42 levels in hippocampus (300μg in total protein) of *LSL-APPswe* mice injected with AAV9-CamkII-GFP or AAV9-CamkII-Cre or Tg2576 mice by Elisa assay. *$p < 0.05$, ***$p < 0.001$, n=3, one-way ANOVA followed by Tukey post hoc test. **(h)** EPMT: Representative tracing images and quantifications of the open arm duration time and entries, n=10 mice. **(i)** LDT: Quantifications of the time spent in the light room and the number of transitions into the light room, n=10. **(j)** TST, **(k)** FST, and **(i)** SPT quantification analyses. All data were presented as mean ± SD, and no significant difference was detected in **h-l** by student's t test.

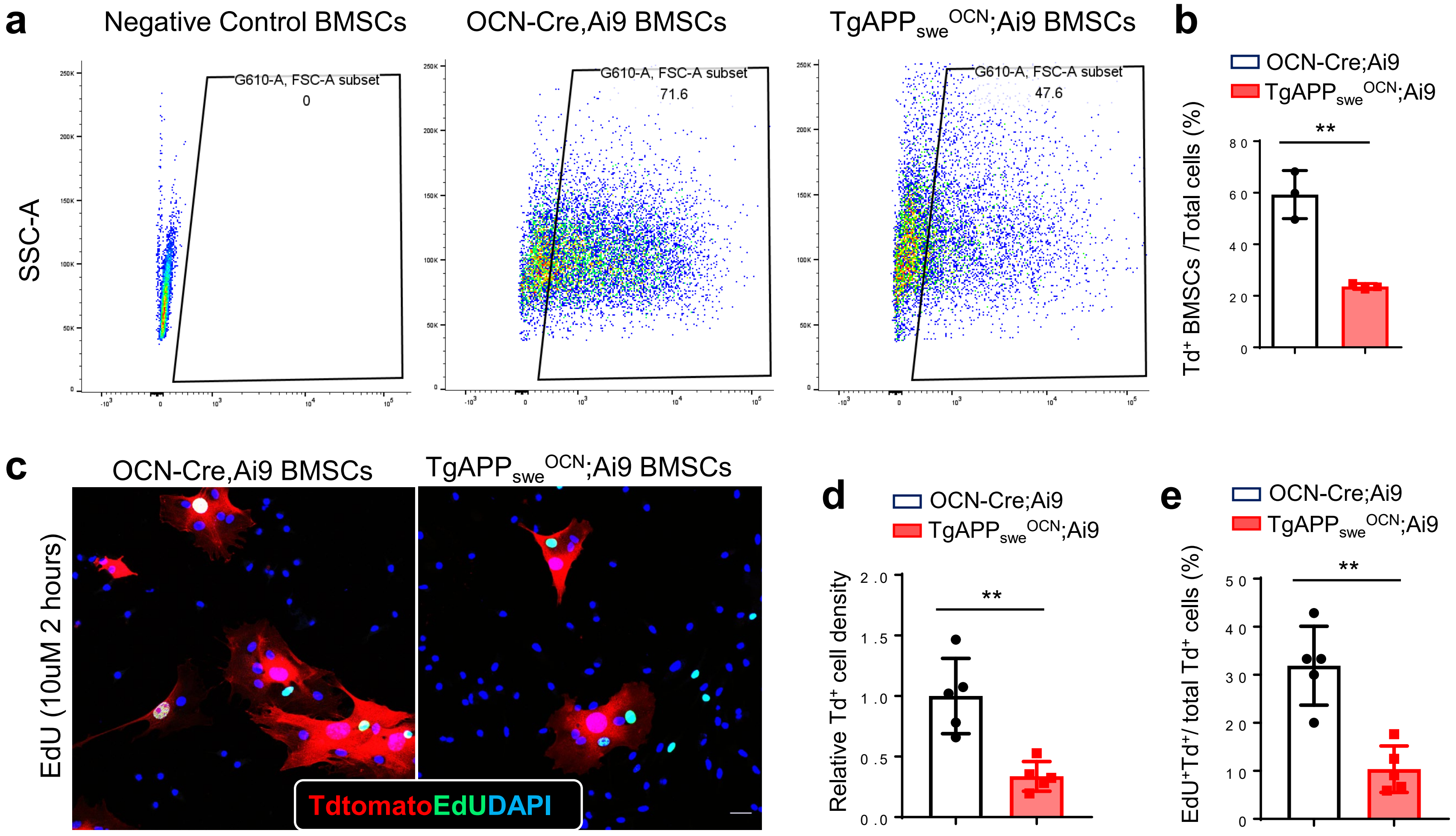
a
Negative Control BMSCs
OCN-Cre,Ai9 BMSCs
TgAPPswe OCN;Ai9 BMSCs
SSC-A
G610-A, FSC-A subset
0
71.6
47.6
b
OCN-Cre;Ai9
TgAPPswe OCN;Ai9
Td+ BMSCs /Total cells (%)
**
c
OCN-Cre,Ai9 BMSCs
TgAPPswe OCN;Ai9 BMSCs
EdU (10uM 2 hours)
TdtomatoEdUDAPI
d
OCN-Cre;Ai9
TgAPPswe OCN;Ai9
Relative Td+ cell density
**
e
OCN-Cre;Ai9
TgAPPswe OCN;Ai9
EdU+Td+/ total Td+ cells (%)
**

Supplementary Fig. 9: Decreased proliferation in 6-MO *TgAPP*$_{swe}^{OCN}$ OB progenitors.

(a-b) FACS analysis of Tdtomato^{+} (Td^{+}) cells in cultured OB progenitors from 6-MO *OCN-Cre; Ai9,* and *TgAPP*$_{swe}^{OCN}$*; Ai9* mice (6-MO). Representative FACS data were shown in **a**. The quantification analysis was presented in **b**. (Mean ± SD, $n = 3$ from 3 different cultures or mice. $^{**}P < 0.01$). **(c-e)** Reductions in OB-progenitor cell proliferation in *TgAPP*$_{swe}^{OCN}$ BMSC culture. BMSCs were incubated with EdU (10 μM, 2 h) and then subjected to EdU kit staining. **c**, Representative images, Scale bar, 20 μm. **d-e**, Td^{+} cell density (**d**) and EdU^{+} Td^{+} cells over total Td^{+} cells(**e**) were quantified (mean ± SD, $n = 5$ from 5 different cultures or mice), $^{**}p < 0.01$, by student's t test.

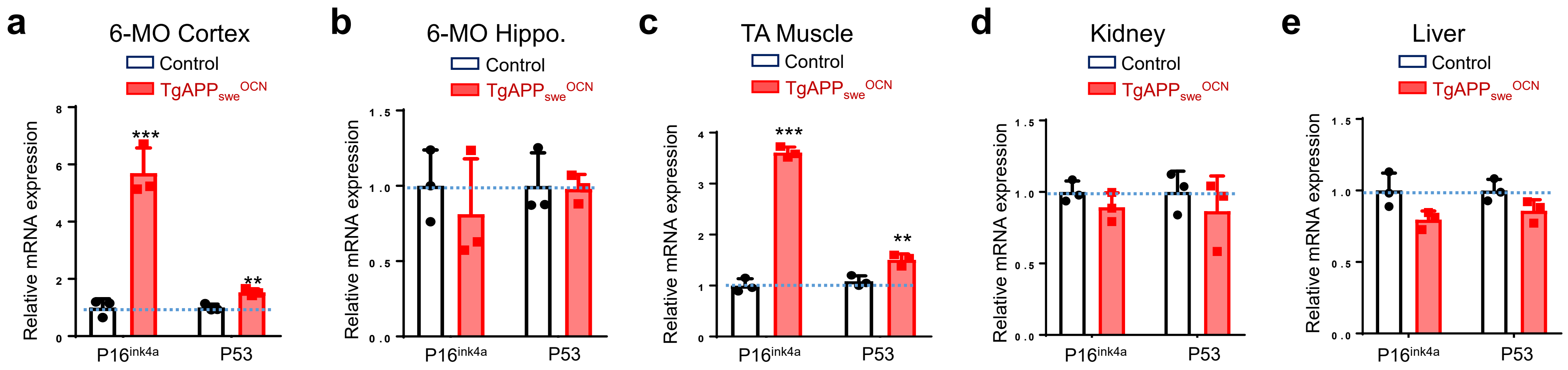

Supplementary Fig. 10: Increased cellular senescence marker, P16^{Ink4a} and P53, in muscle and cortex of 6-MO *TgAPP$_{swe}^{OCN}$* mice.

(a-e) RT-PCR analysis of P16^{Ink4a}, P53 gene expression in the cortex(**a**), hippocampus (**b**), the TA (tibialis anterior) muscle(**c**), kidney (**d**) and liver (**e**) of 6-MO control (LSL-APP$_{swe}$) and *TgAPP$_{swe}^{OCN}$* mice. **p<0.01, ***p < 0.001, n=3, Mann-Whitney U test.

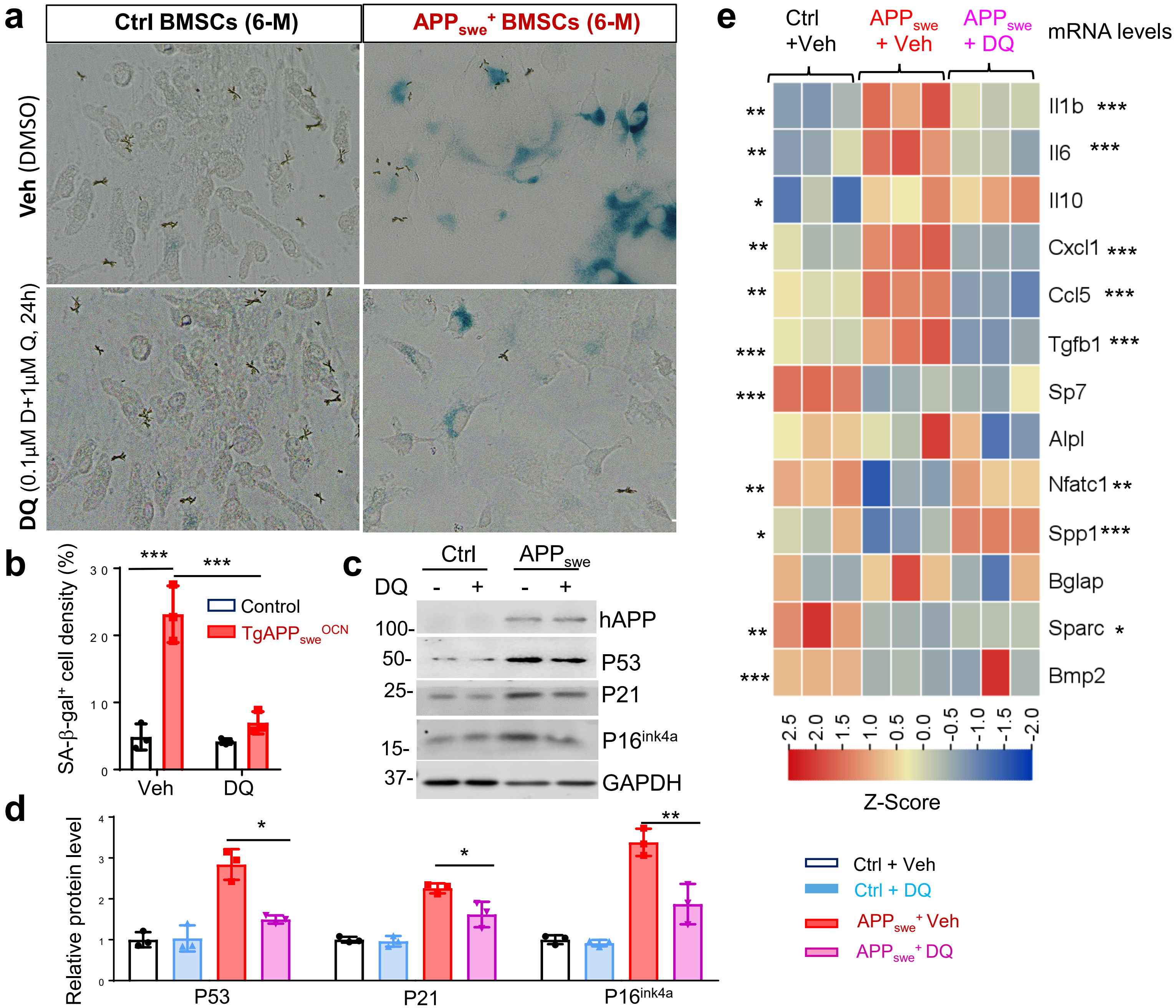

a
Ctrl BMSCs (6-M)
APPswe+ BMSCs (6-M)
Veh (DMSO)
DQ (0.1μM D+1μM Q, 24h)
b
SA-β-gal+ cell density (%)
***
***
Control
TgAPPswe OCN
Veh
DQ
c
Ctrl
APPswe
DQ - + - +
hAPP
P53
P21
P16ink4a
GAPDH
100-
50-
25-
15-
37-
d
Relative protein level
P53
P21
P16ink4a
e
Ctrl +Veh
APPswe + Veh
APPswe + DQ
mRNA levels
Il1b ***
Il6 ***
Il10
Cxcl1 ***
Ccl5 ***
Tgfb1 ***
Sp7
Alpl
Nfatc1 **
Spp1***
Bglap
Sparc *
Bmp2
Z-Score
Ctrl + Veh
Ctrl + DQ
APPswe+ Veh
APPswe+ DQ

Supplementary Fig. 11: Abolished increases of cellular senescence in 6-MO *$TgAPP_{swe}^{OCN}$* BMSCs by treatment with senescence inhibitors, D + Q.

(a) SA-β-gal staining of 6-MO control and *$TgAPP_{swe}^{OCN}$* BMSCs *with* vehicle (Veh-DMSO) treatment and 0.1μM Dasatinib(D) + 1μM Quercetin (Q) treatment respectively, scale bar, 20μm. (**b**) Quantification of SA-β-gal^{+} cell densities in **a** (mean ± SD; n=3). **(c)** Western blot analysis of indicated protein expression in BMSCs from 6-MO control and *$TgAPP_{swe}^{OCN}$* with Veh or DQ treatment. **(d)** Quantification analyses of the data in **c**, *p<0.05, n=3. **(e)** RT-PCR analysis of the indicated gene expression in control BMSCs with Veh treatment, *$TgAPP_{swe}^{OCN}$* BMSCs with Veh or DQ treatment. Left significance analyses were from comparison between control BMSCs with Veh treatment and *$TgAPP_{swe}^{OCN}$* BMSCs with Veh treatment. Right significance analyses were from comparison between *$TgAPP_{swe}^{OCN}$* BMSCs with Veh and *$TgAPP_{swe}^{OCN}$* with DQ treatment. *p<0.05, **p<0.01, ***p<0.001. n=3, and data are presented as heatmap. Two-way analysis of variance test was used in **b** and **d**; one-way ANOVA followed by Tukey post hoc test was used in **e**. Data in **b** and **d** were shown as mean ± SD.

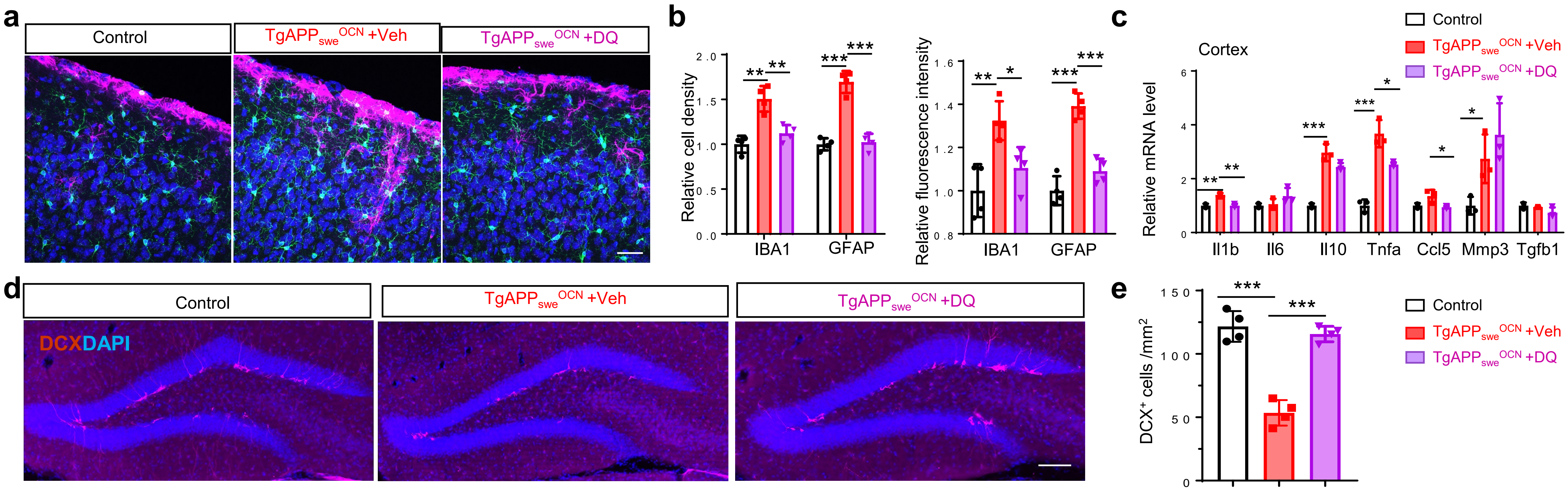
a
Control
TgAPPswe OCN +Veh
TgAPPswe OCN +DQ
b
Relative cell density
IBA1
GFAP
Relative fluorescence intensity
c
Cortex
Relative mRNA level
Il1b
Il6
Il10
Tnfa
Ccl5
Mmp3
Tgfb1
Control
TgAPPswe OCN +Veh
TgAPPswe OCN +DQ
d
DCX DAPI
e
DCX+ cells /mm2

Supplementary Fig. 12: Attenuated gliosis and neurogenesis deficit in $TgAPP_{swe}^{OCN}$ mice treated with senescence inhibitor.

(a) Representative images of cortical brain sections from 6.5-MO control (*LSL-*APP_{swe}) and $TgAPP_{swe}^{OCN}$ with Veh or DQ treatments coimmunostained with IBA1 (green), GFAP (magenta), and DAPI (blue). Scale bar, 20μm. **(b)** Quantification analyses of the GFAP and IBA1 fluorescence intensity and cell densities of data in **a.** *p<0.05, **p<0.01, ***p < 0.001. n=4. **(c)** RT-PCR analysis of indicated gene expression in the cortex of 6.5-MO control and $TgAPP_{swe}^{OCN}$ mice with Veh or DQ treatments. *p<0.05, **p<0.01, ***p < 0.001, n=3 mice in each group. (**d**) Representative images of dorsal DG from 6.5-MO control and $TgAPP_{swe}^{OCN}$ mice coimmunostained with DCX (magenta) and DAPI (blue). Scale bar, 100μm. **(e)** Quantification analyses of DCX^+ cell densities in dorsal DG of data in **d**, n=4, ****P* < 0.001. All the data were presented as mean ± SD, and one-way ANOVA followed by Tukey post hoc test.

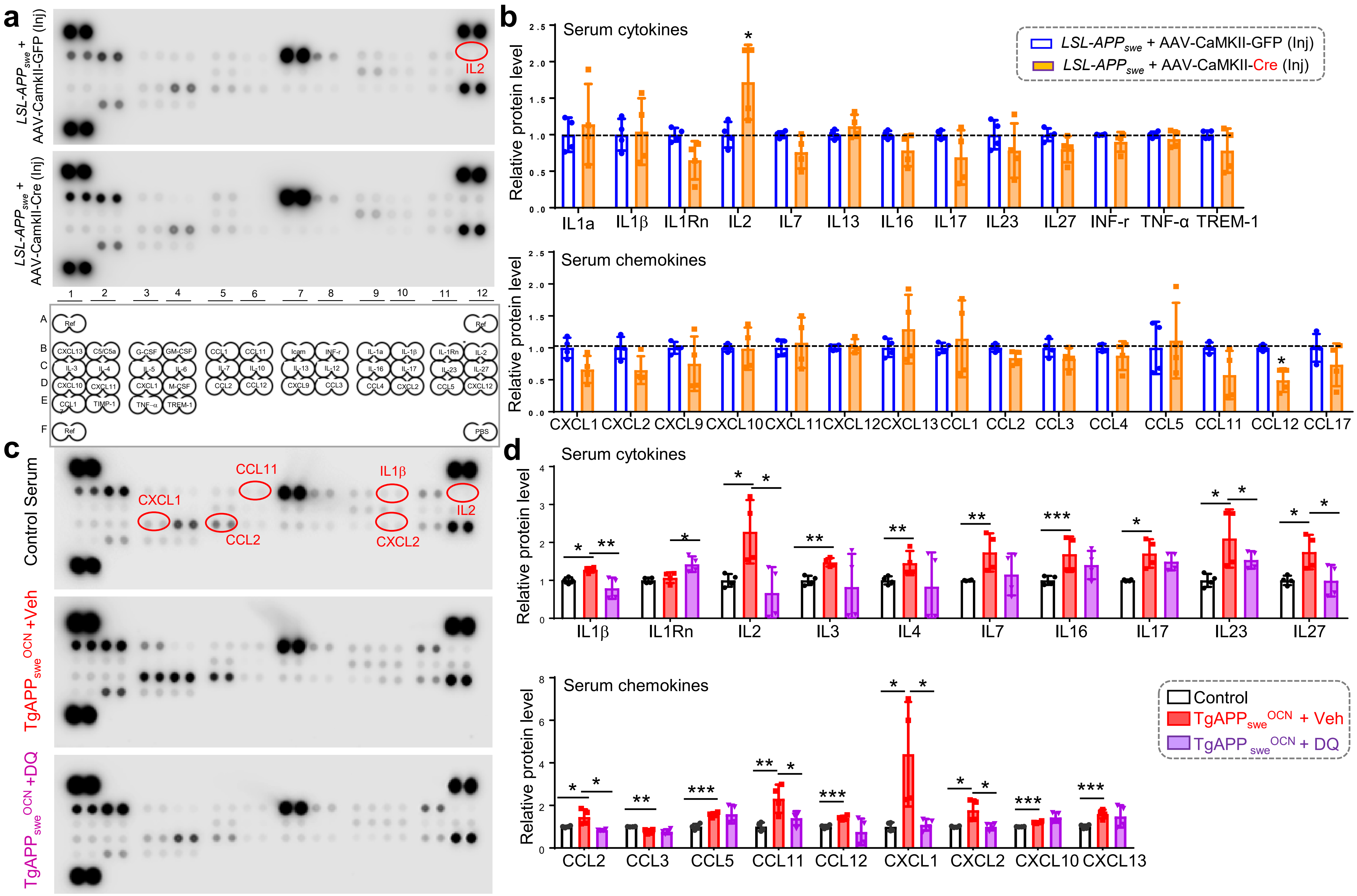
a
LSL-APPswe + AAV-CamkII-GFP (Inj)
LSL-APPswe + AAV-CamkII-Cre (Inj)
IL2
1 2 3 4 5 6 7 8 9 10 11 12
A B C D E F
Ref
CXCL13 C5/C5a G-CSF GM-CSF CCL1 CCL11 Icam INF-r IL-1a IL-1β IL-1Rn IL-2
IL-3 IL-4 IL-5 IL-6 IL-7 IL-10 IL-13 IL-12 IL-16 IL-17 IL-23 IL-27
CXCL10 CXCL11 CXCL1 M-CSF CCL2 CCL12 CXCL9 CCL3 CCL4 CXCL2 CCL5 CXCL12
CCL17 TIMP-1 TNF-α TREM-1
PBS
b
Serum cytokines
LSL-APPswe + AAV-CaMKII-GFP (Inj)
LSL-APPswe + AAV-CaMKII-Cre (Inj)
Relative protein level
IL1a IL1β IL1Rn IL2 IL7 IL13 IL16 IL17 IL23 IL27 INF-r TNF-α TREM-1
Serum chemokines
CXCL1 CXCL2 CXCL9 CXCL10 CXCL11 CXCL12 CXCL13 CCL1 CCL2 CCL3 CCL4 CCL5 CCL11 CCL12 CCL17
c
Control Serum
TgAPPswe OCN +Veh
TgAPPswe OCN +DQ
CXCL1 CCL11 IL1β IL2 CCL2 CXCL2
d
Serum cytokines
Relative protein level
IL1β IL1Rn IL2 IL3 IL4 IL7 IL16 IL17 IL23 IL27
Serum chemokines
Control
TgAPPswe OCN + Veh
TgAPPswe OCN + DQ
CCL2 CCL3 CCL5 CCL11 CCL12 CXCL1 CXCL2 CXCL10 CXCL13

Supplementary Fig. 13: Attenuated SASPs in *TgAPP*$_{swe}$^{OCN} mice treated with senescence inhibitors.

(a) Proteome profile of Mouse Cytokine Array of *LSL-APPswe* mice injected with AAV9-CamkII-GFP or AAV9-CamkII-Cre serum. **(b)** Quantification analyses of the data in **a**, *p<0.05, n=4. **(c)** Proteome profile of Mouse Cytokine Array of 6.5-MO control and *TgAPP*$_{swe}$^{OCN} mouse (male) serum with Veh or DQ treatments. **(d)** Quantification analyses of the data in **c**, *p<0.05, **p<0.01, ***p < 0.001. n=4. The values were presented as mean ± SD. One-way ANOVA followed by Tukey post hoc test.

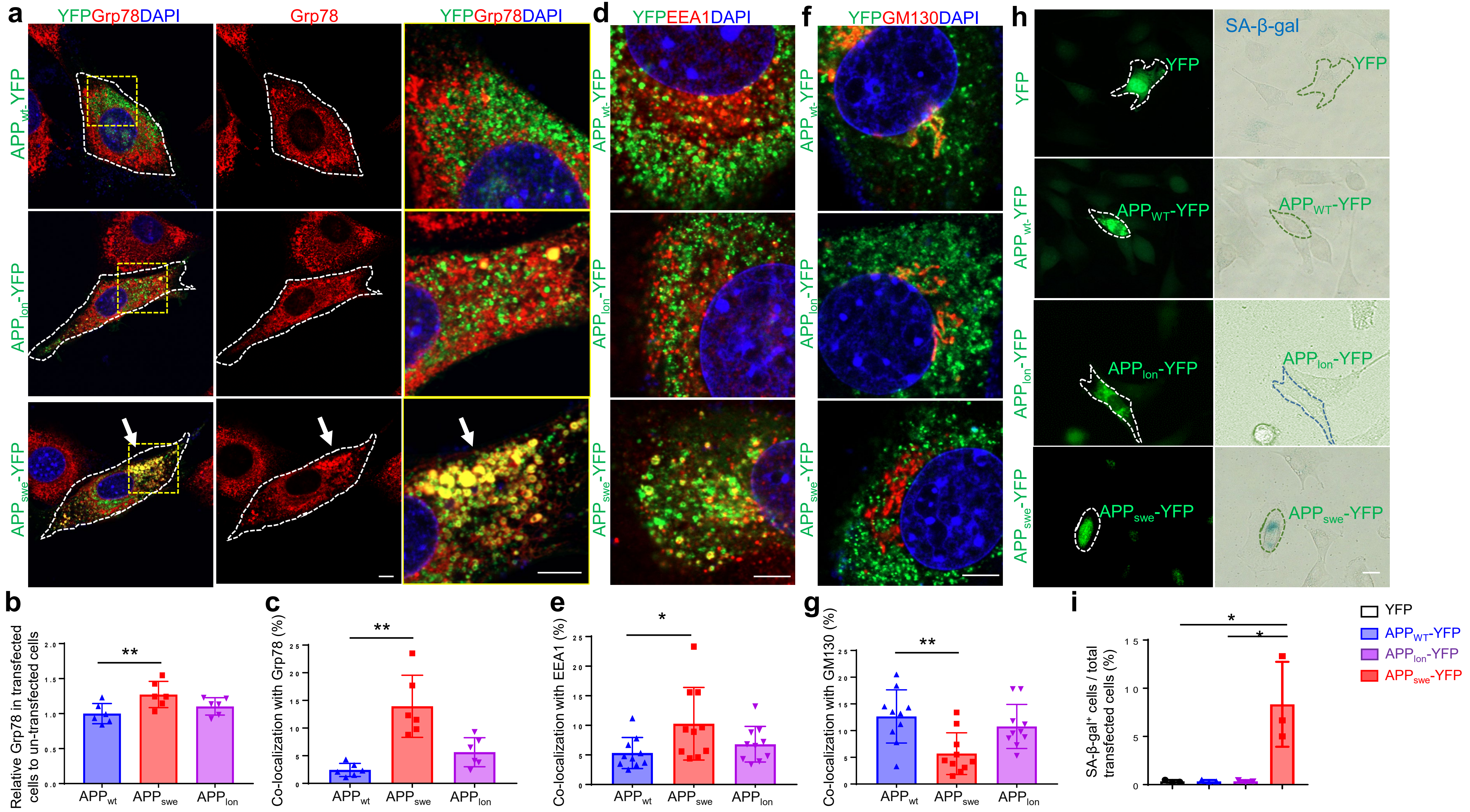

a
YFPGrp78DAPI
Grp78
YFPGrp78DAPI
APPwt-YFP
APPlon-YFP
APPswe-YFP
d
YFPEEA1DAPI
f
YFPGM130DAPI
h
SA-β-gal
YFP
APPWT-YFP
APPlon-YFP
APPswe-YFP
b
Relative Grp78 in transfected cells to un-transfected cells
**
APPwt
APPswe
APPlon
c
Co-localization with Grp78 (%)
**
e
Co-localization with EEA1 (%)
*
g
Co-localization with GM130 (%)
**
i
SA-β-gal+ cells / total transfected cells (%)
*
*
YFP
APPWT-YFP
APPlon-YFP
APPswe-YFP

Supplementary Fig. 14: Increased SA-β-gal staining in MC3T3 cells (an OB cell line) expressing APP_{swe}-YFP, but not APP_{WT}-YFP or APP_{lon}-YFP.

(a) Representative images of MC3T3 cells transfected with APP_{wt}-YFP, APP_{swe}-YFP and APP_{lon}-YFP plasmids coimmunostained with Grp78(red) and DAPI (blue). Scale bar, 5μm. **(b-c)** Quantification analyses of relative Grp78 fluorescence intensity in transfected cells to un-transfected cells (fold over APP_{wt}) (**b**) and percentage of YFP co-localization with Grp78 in total YFP(**c**), **p<0.01. **(d)** Representative images of MC3T3 cells transfected with APP_{wt}-YFP, APP_{swe}-YFP and APP_{lon}-YFP plasmids coimmunostained with EEA1(red) and DAPI (blue). Scale bar, 5μm. **(e)** Quantification analyses of percentage of YFP co-localization with EEA1 in total YFP, *p<0.05. **(f)** Representative images of MC3T3 cells transfected with APP_{wt}-YFP, APP_{swe}-YFP and APP_{lon}-YFP plasmids coimmunostained with GM130(red) and DAPI (blue). Scale bar, 5μm. **(g)** Quantification analyses of percentage of YFP co-localization with GM130 in total YFP, **p<0.01. (**h**) SA-β-gal staining of MC3T3 cells transfected with YFP, APP_{swe}-YFP, APP_{WT}-YFP and APP_{lon}-YFP plasmids. Scale bar, 20μm. (**i**) Quantification of SA-β-gal^{+} cell densities of data in **h** (n=3). *p<0.05. All the data were presented as mean ± SD, and one-way ANOVA followed by Tukey post hoc test.

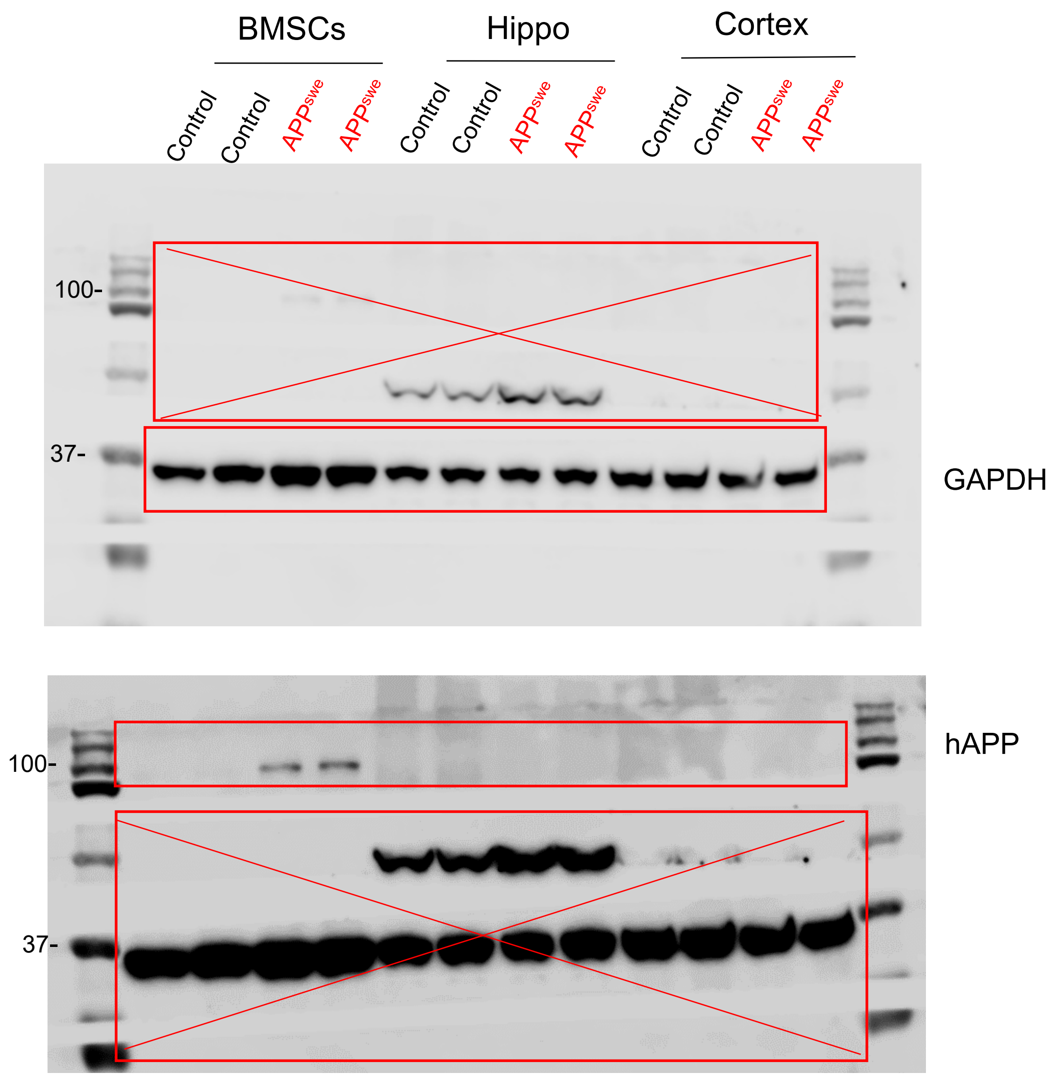

Supplementary Fig. 15: Uncropped blots referring to Fig. 1b.

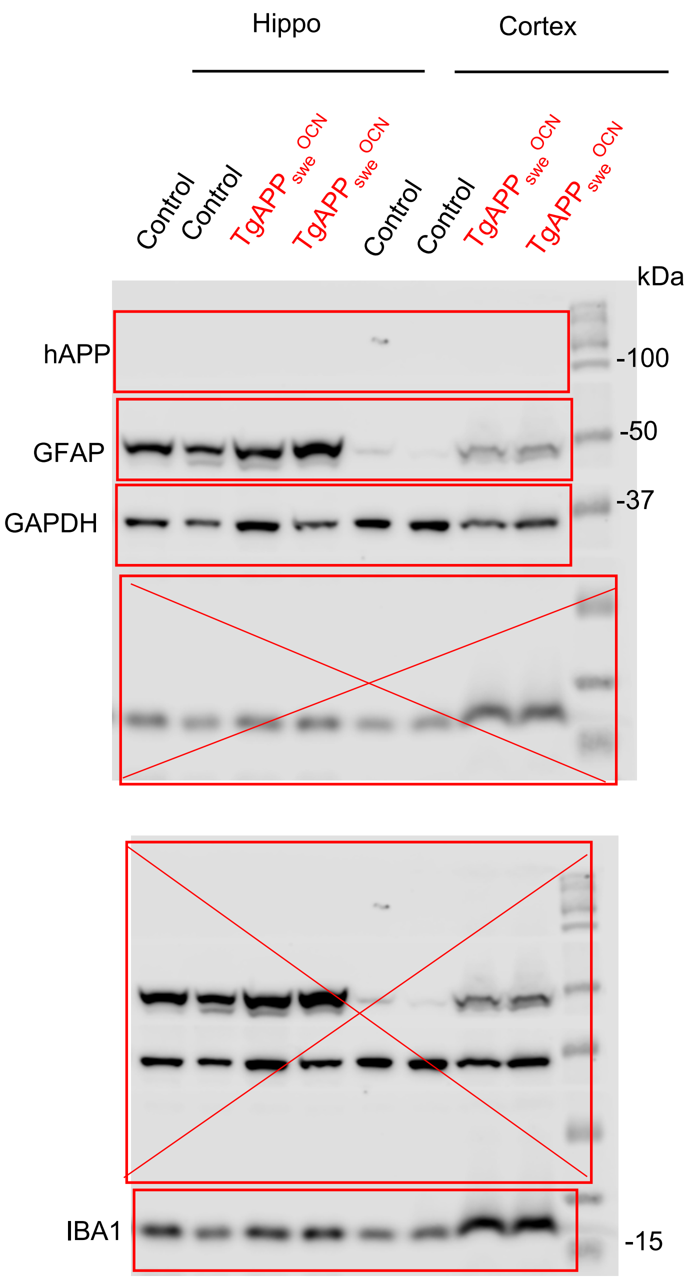

Supplementary Fig. 16: Uncropped blots referring to Fig. 2e.

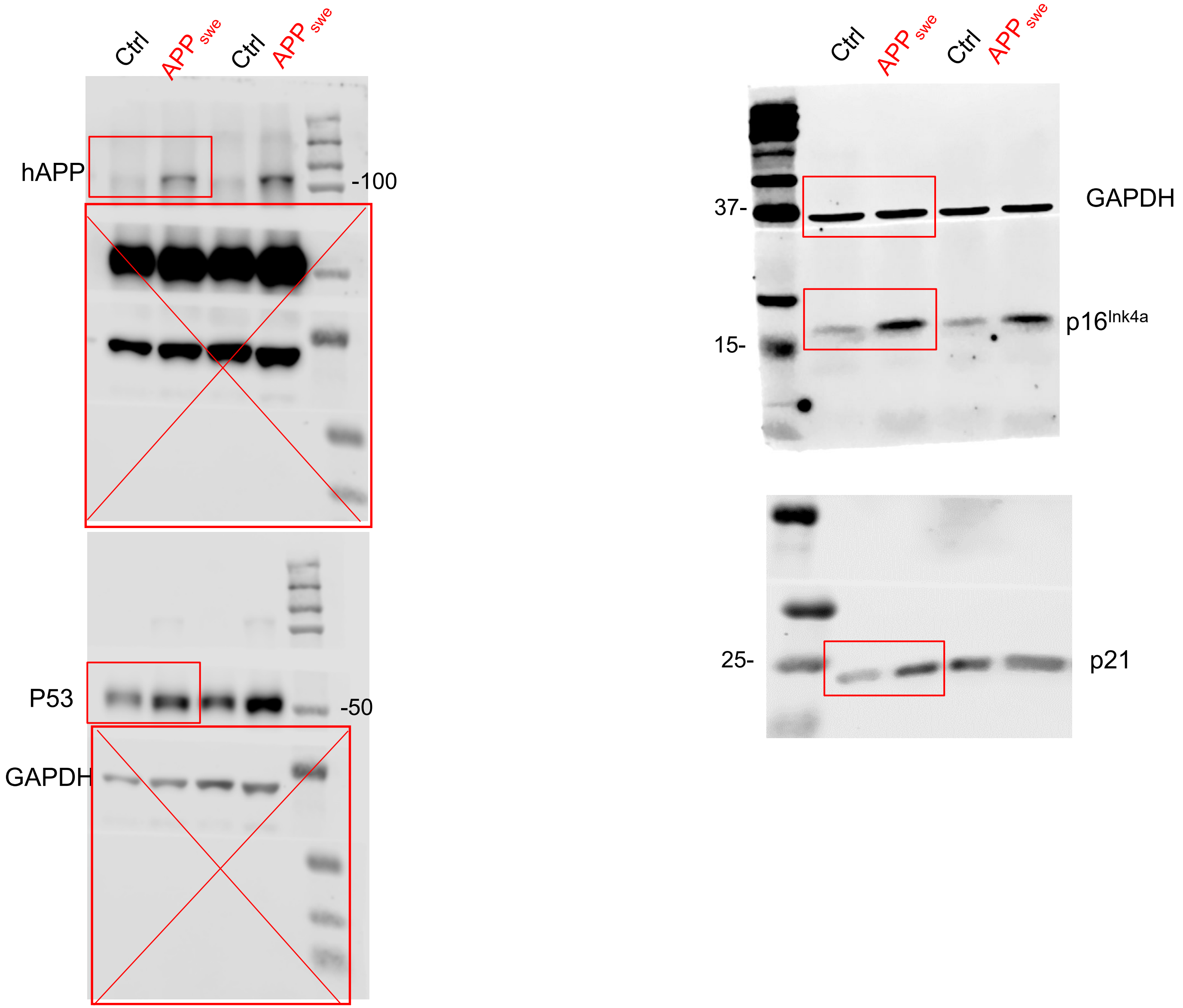

Supplementary Fig. 17: Uncropped blots referring to Fig. 6c.

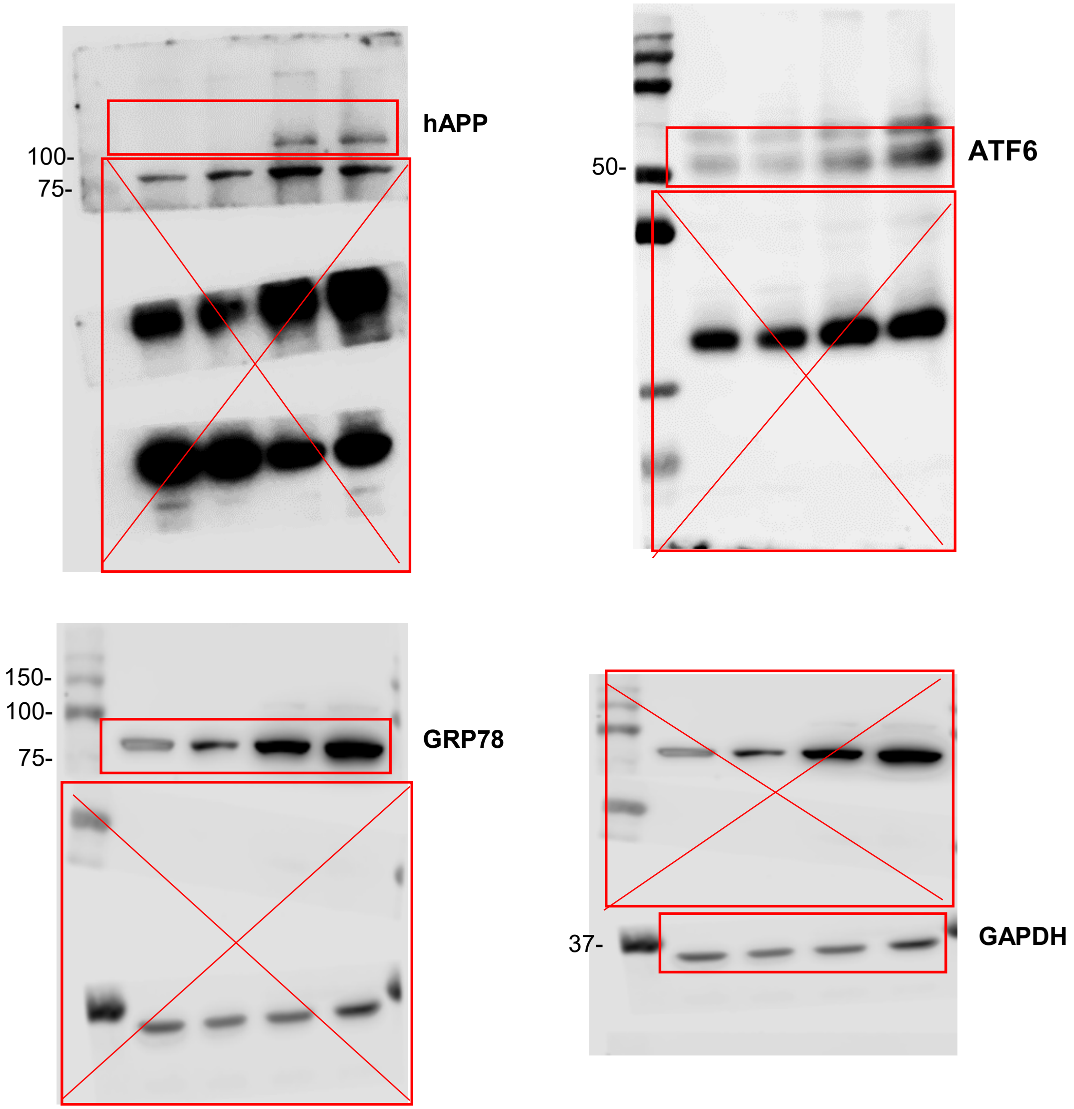

Supplementary Fig. 18: Uncropped blots referring to Fig. 9c.

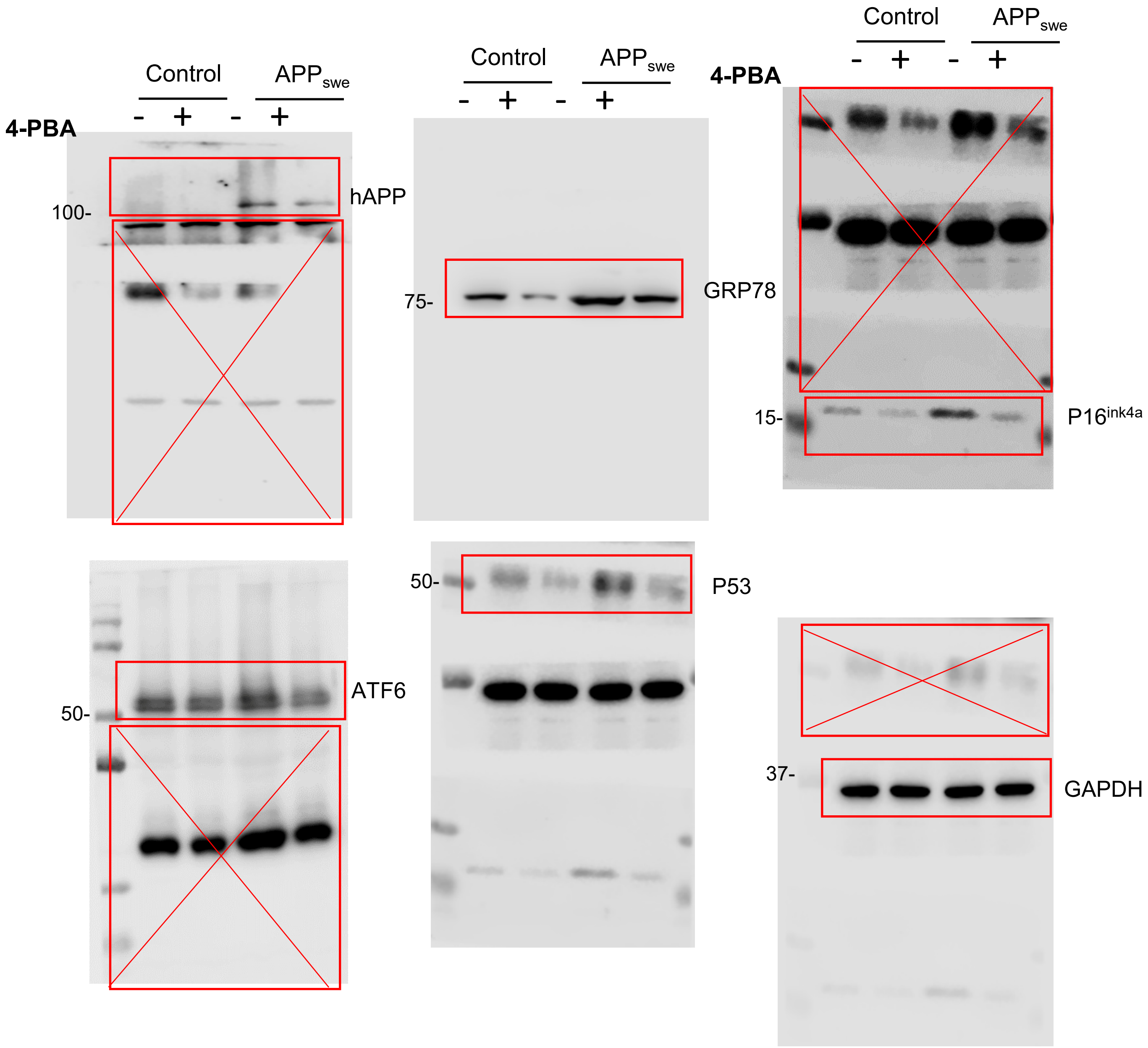

Supplementary Fig. 19: Uncropped blots referring to Fig. 9e.

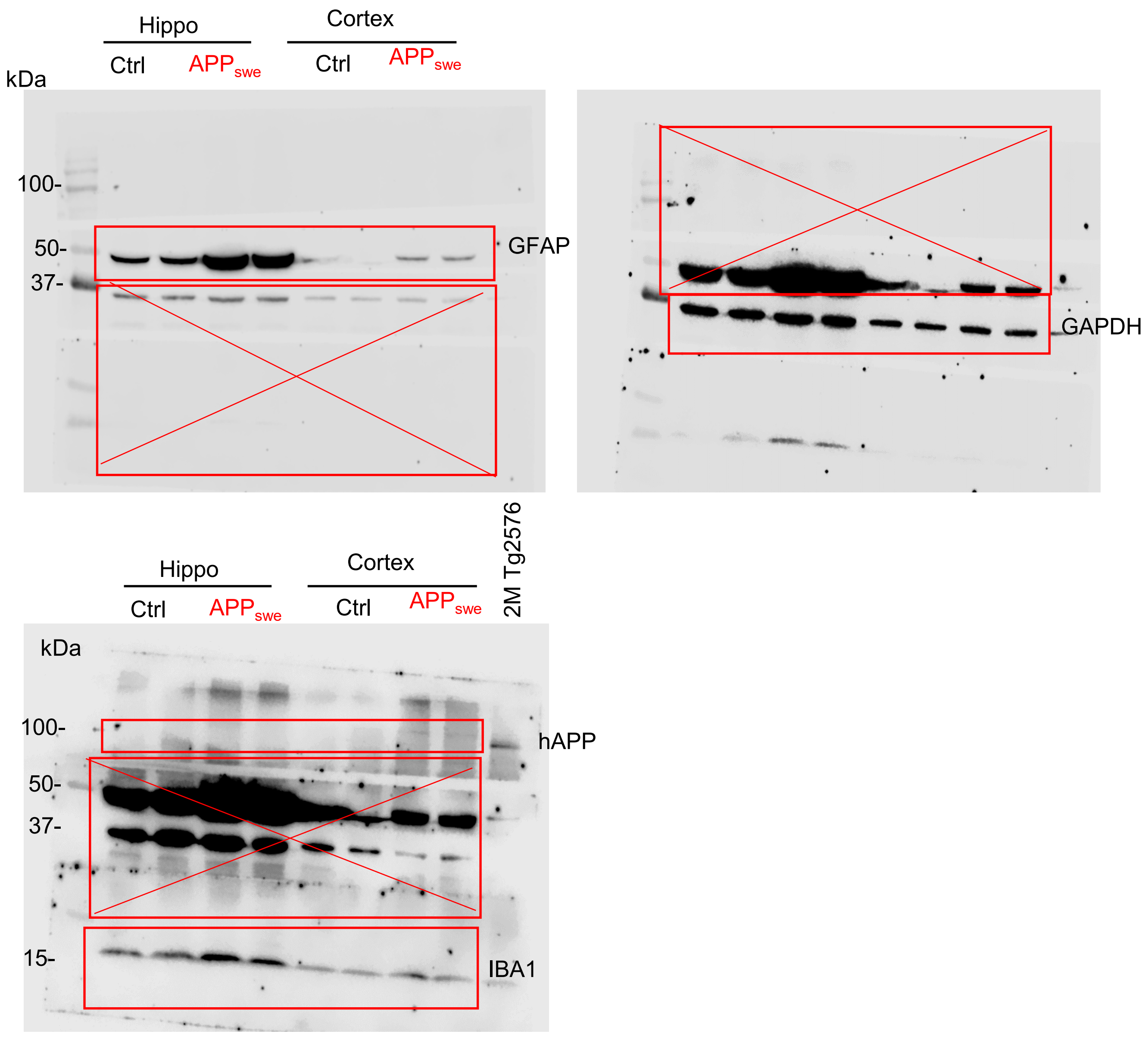

Supplementary Fig. 20: Uncropped blots referring to Supplementary Fig. 5e.

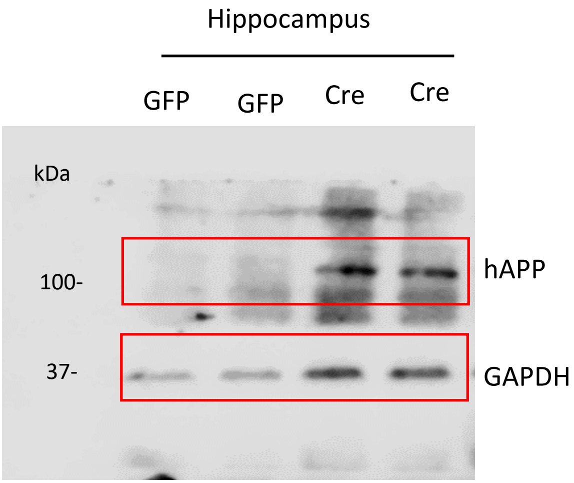

Supplementary Fig. 21: Uncropped blots referring to Supplementary Fig. 8d.

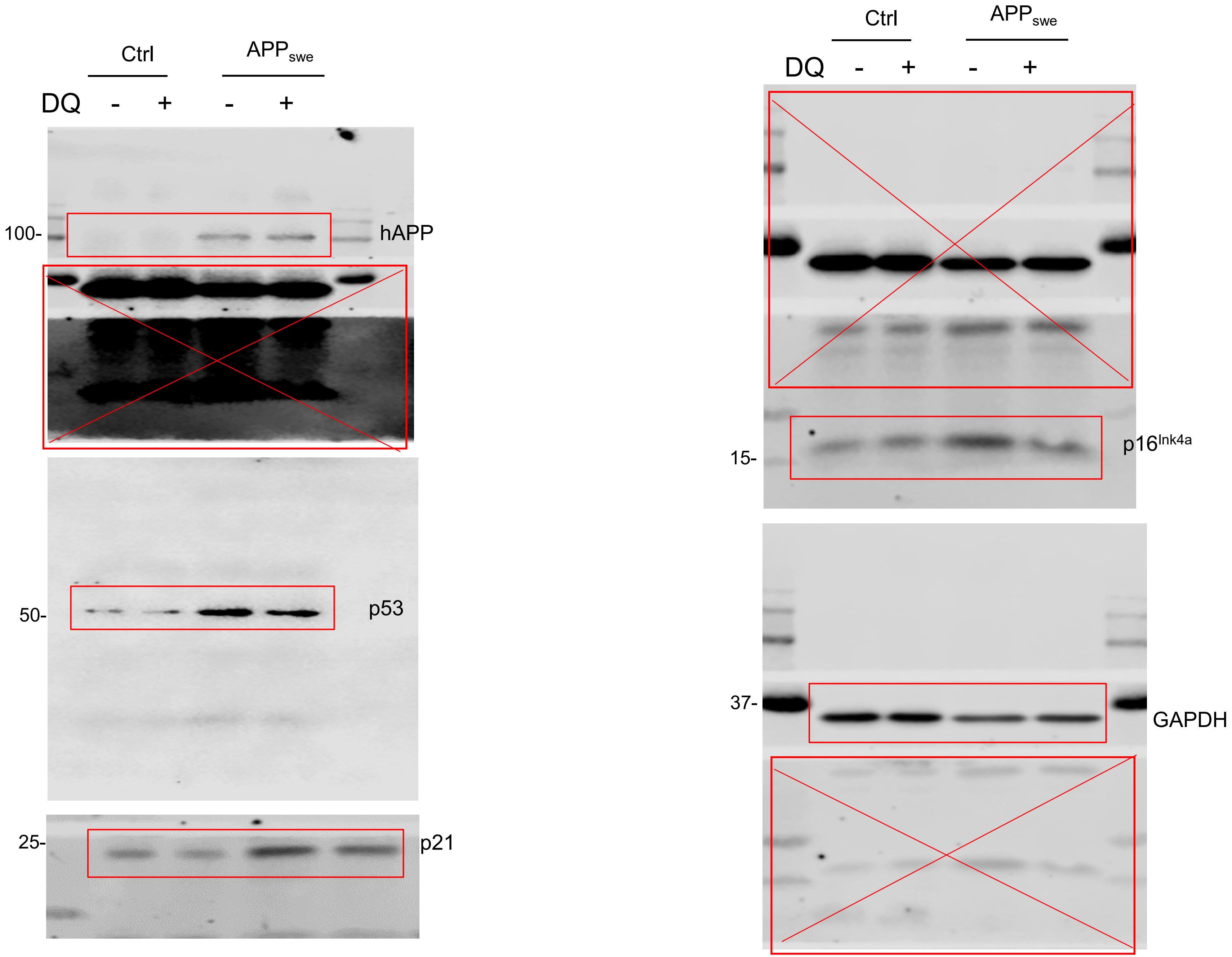

Supplementary Fig. 22: Uncropped blots referring to Supplementary Fig. 11c.
