# Supplementary material for: Osteoblastic Swedish mutant APP expedites brain deficits by inducing endoplasmic reticulum stress-driven senescence
Source: Commun Biol. 2021 Nov 25;4:1326. doi: 10.1038/s42003-021-02843-2 (PMC8617160; doi:10.1038/s42003-021-02843-2)
Supplement: Supplementary file 3 — Description of Additional Supplementary Files [file 42003_2021_2843_MOESM3_ESM.pdf]

Description of Additional Supplementary Files

File name: Supplementary Data 1

Description: Source data underlying Figs. 1-9.
