# Supplementary material for: Osteoblastic Swedish mutant APP expedites brain deficits by inducing endoplasmic reticulum stress-driven senescence
Source: Commun Biol. 2021 Nov 25;4:1326. doi: 10.1038/s42003-021-02843-2 (PMC8617160; doi:10.1038/s42003-021-02843-2)
Supplement: Supplementary file 5 — Reporting Summary [file 42003_2021_2843_MOESM5_ESM.pdf]

nature research

Corresponding author(s): Wen-Cheng Xiong

Last updated by author(s): Oct 11, 2021

Reporting Summary

Nature Research wishes to improve the reproducibility of the work that we publish. This form provides structure for consistency and transparency in reporting. For further information on Nature Research policies, see our Editorial Policies and the Editorial Policy Checklist.

Statistics

For all statistical analyses, confirm that the following items are present in the figure legend, table legend, main text, or Methods section.

n/a	Confirmed	
☐	☒	The exact sample size (n) for each experimental group/condition, given as a discrete number and unit of measurement
☐	☒	A statement on whether measurements were taken from distinct samples or whether the same sample was measured repeatedly
☐	☒	The statistical test(s) used AND whether they are one- or two-sided<br>*Only common tests should be described solely by name; describe more complex techniques in the Methods section.*
☒	☐	A description of all covariates tested
☐	☒	A description of any assumptions or corrections, such as tests of normality and adjustment for multiple comparisons
☐	☒	A full description of the statistical parameters including central tendency (e.g. means) or other basic estimates (e.g. regression coefficient) AND variation (e.g. standard deviation) or associated estimates of uncertainty (e.g. confidence intervals)
☐	☒	For null hypothesis testing, the test statistic (e.g. F, t, r) with confidence intervals, effect sizes, degrees of freedom and P value noted<br>*Give P values as exact values whenever suitable.*
☒	☐	For Bayesian analysis, information on the choice of priors and Markov chain Monte Carlo settings
☒	☐	For hierarchical and complex designs, identification of the appropriate level for tests and full reporting of outcomes
☒	☐	Estimates of effect sizes (e.g. Cohen's d, Pearson's r), indicating how they were calculated

Our web collection on statistics for biologists contains articles on many of the points above.

Software and code

Policy information about availability of computer code

Data collection	All images raw data were collected by image J software, Deseq2 and PossionDis algorithms were used to detect differential expression genes for RNA-seq.
Data analysis	Graphs were generated by GraphPad 7.0 , RNA-Seq data analysis by R version 4.0

For manuscripts utilizing custom algorithms or software that are central to the research but not yet described in published literature, software must be made available to editors and reviewers. We strongly encourage code deposition in a community repository (e.g. GitHub). See the Nature Research guidelines for submitting code & software for further information.

Data

Policy information about availability of data

All manuscripts must include a data availability statement. This statement should provide the following information, where applicable:

- Accession codes, unique identifiers, or web links for publicly available datasets
- A list of figures that have associated raw data
- A description of any restrictions on data availability

We declare that all data supporting the findings of this study are available within the article and its source data, RNA-seq data are included in source data file

Field-specific reporting

Life sciences study design

All studies must disclose on these points even when the disclosure is negative.

Sample size	We used Power analysis to decide the sample size, If we have two different samples test statistics = Z_a and Z-b with a significance level of a = .05 and standard deviation = d and standard error = s. We calculate sample size, n, as: n=(s^2 (Z_a+Z_b)^2)/d^2
Data exclusions	No data was excluded.
Replication	All experiments were repeated multiple times (at least 3) and the results were found to be reproducible
Randomization	Animals were randomized into the treatment groups
Blinding	Blinding was used in Animal behavior tests

Reporting for specific materials, systems and methods

We require information from authors about some types of materials, experimental systems and methods used in many studies. Here, indicate whether each material, system or method listed is relevant to your study. If you are not sure if a list item applies to your research, read the appropriate section before selecting a response.

Materials & experimental systems

n/a	Involved in the study	
☐	☒	Antibodies
☐	☒	Eukaryotic cell lines
☒	☐	Palaeontology and archaeology
☐	☒	Animals and other organisms
☒	☐	Human research participants
☒	☐	Clinical data
☒	☐	Dual use research of concern

Methods

n/a	Involved in the study	
☒	☐	ChIP-seq
☐	☒	Flow cytometry
☒	☐	MRI-based neuroimaging

Antibodies

Antibodies used	Anti-hAPP (6E10, 803001, mouse) and anti-6E10(Alexa Fluor® 647 anti-β-Amyloid, 1-16 Antibody, cat#803021) from biolegend (San Diego, California, USA); Anti-Amyloid Fibrils OC antibody (AB2286, rabbit) from EMD Millipore(Temecula, California, USA); Anti-DCX (SC-8066, goat) from Santa Cruz Biotech (Santa Cruz, California, USA); Anti-Ctip2 (ab18465, Rat), anti-IBA1 (ab178846, rabbit and ab5076, goat), anti-P16ink4a (ab211542, rabbit), and anti-P53 (ab26, mouse) from Abcam (Cambridge, Massachusetts, USA); Anti-S100β (287004, Guinea pig) from Synaptic System (Göttingen, Germany); Anti-Olig2 (p21954, rabbit), anti-GRP78 (PA1-014A, rabbit) and anti-EEA1 (PA1-063A, rabbit) from Invitrogen (Carlsbad, California, USA); Anti-ATF6 (NBP1-40256, mouse) from Novus biologicals (Centennial, CO, USA); anti-GM130 (610822, mouse) from BD biosciences (San Jose, CA, USA) and Anti-P21 (2947S, rabbit), anti-NEUN) (12943S, rabbit), anti-GFAP (12389S, rabbit), and anti-GAPDH (97166S, mouse) from cell signaling (Danvers, Massachusetts, USA). Secondary antibodies were purchased from Jackson ImmunoResearch Laboratories (West Grove, Pennsylvania, USA).
Validation	Antibodies were used according to the manufacturers' specifications

Eukaryotic cell lines

Policy information about cell lines

Cell line source(s)	MC3T3 cell line was Mus musculus (mouse) calvaria
Authentication	MC3T3 cell line was purchased from ATCC
Mycoplasma contamination	All cell lines tested negative for mycoplasma
Commonly misidentified lines (See ICLAC register)	N/A

Animals and other organisms

Policy information about studies involving animals; ARRIVE guidelines recommended for reporting animal research

Laboratory animals	The LSL-APPswe mice were generated by our lab, The OCN–Cre mice were kindly provided by Tom Clemens (Johns Hopkins Medical School). Ai9 mice was purchase from the Jackson Laboratory, The Tg2576 mice were purchased from Taconic, Hudson, NY, USA.

	5xFAD transgenic mice were obtained from The Jackson Laboratory (MMRRC stock #34 840-JAX). All mouse lines were backcrossed into C57BL/6 background and housed in a room with a 12 h light/dark cycle and ad libitum access to water and rodent chow diet (Harlan Tekled S-2335).
Wild animals	Study did not involve wild animals
Field-collected samples	Study did not involve samples collected from the field
Ethics oversight	All experimental procedures on mice were approved by the Institutional Animal Care and Use Committee at Case Western Reserve University (IACUC, 2017–0121), according to the United States National Institutes of Health guidelines.

Note that full information on the approval of the study protocol must also be provided in the manuscript.

Flow Cytometry

Plots

Confirm that:

- [x] The axis labels state the marker and fluorochrome used (e.g. CD4-FITC).
- [x] The axis scales are clearly visible. Include numbers along axes only for bottom left plot of group (a 'group' is an analysis of identical markers).
- [x] All plots are contour plots with outliers or pseudocolor plots.
- [x] A numerical value for number of cells or percentage (with statistics) is provided.

Methodology

Sample preparation	BMSCs were cultured of 6-MO OCN-Cre; Ai9 and TgAPPsweOCN; Ai9 mice, the attached Bone marrow cells were cultured with the growth medium for 7 days. These cells were then resuspended and plated at 100mm culture dishes, and cultured for another 3 days with the same growth medium. Then cells were detached from culture dishes by Trypsin solution. the detached adherent cells were centrifuged and the pellet cells were washed with 1 ml cold PBS, and finally resuspended in 0.5 ml PBS with 1% FBS for flow cytometry analysis.
Instrument	BD Biosciences Aria
Software	FACSDiva 8.0.1 software (BD)
Cell population abundance	30-80% cells in total cells were collected based on Tdtomato experssion
Gating strategy	Using Wild type BMSCs without Tdtomato-expression as negative control

- [x] Tick this box to confirm that a figure exemplifying the gating strategy is provided in the Supplementary Information.
